# Reshaping of soil properties and microbial community by the conversion from non-grain cultivated land to paddy field

**DOI:** 10.3389/fmicb.2025.1643144

**Published:** 2025-08-29

**Authors:** Xuqing Li, Han Chen, Xiao Wang, Qurban Ali, Luqiong Lv, Tiefeng Zhou, Munazza Ijaz, Temoor Ahmed, Jianli Yan, Bin Li

**Affiliations:** ^1^Institute of Vegetable, Hangzhou Academy of Agricultural Sciences, Hangzhou, China; ^2^Ningbo Jiangbei District Agricultural Technology Extension Service Station, Ningbo, China; ^3^Department of Biology, College of Science, United Arab Emirates University, Abu Dhabi, United Arab Emirates; ^4^State Key Laboratory of Rice Biology and Breeding, Ministry of Agriculture Key Laboratory of Molecular Biology of Crop Pathogens and Insects, Zhejiang Key Laboratory of Biology and Ecological Regulation of Crop Pathogens and Insects, Institute of Biotechnology, Zhejiang University, Hangzhou, China; ^5^Department of Life Sciences, Western Caspian University, Baku, Azerbaijan

**Keywords:** non-grain cultivated land, converted paddy fields, soil bacterial community structure, soil metabolite, soil physicochemical properties

## Abstract

**Background:**

In order to ensure food security, China is actively carrying out conversion of nongrain cultivated land to paddy field. Therefore, it is very necessary to investigate the influence of this conversion on soil health, which has been well known to play an important role in crop growth.

**Methods:**

A combined analysis of soil physicochemical properties, bacterial community structure, and metabolite was conducted on 72 soil samples, which were collected in this study from the converted paddy fields and the corresponding non-grain cultivated lands including loquat garden, mulberry field, blueberry garden, vineyard, bamboo garden and nursery stock base.

**Results:**

In this study, conversion of non-grain cultivated land to paddy field significantly influenced physicochemical properties, bacterial community structure, and metabolite of root-zone soil with 8.08–43.85%, 8.90–64.14%, 24.98–91.97%, 38.74–92.52%, and 5.12–32.99% reduction in soil organic matter content (SOM), alkaline hydrolysis nitrogen (AHN), available phosphorus (AP), available potassium (AK), and microbial biomass carbon (MBC), respectively; 0.81–3.08 fold, 1.26–21.50 fold, and 4.29–14.54 fold increase in relative abundance (RAs) of Chloroflexi, Desulfobacterota, and Nitrospirota, respectively; and 2,204 differentially expressed metabolite (DEMs) belonging to amino acids and derivatives, benzene and substituted derivatives, flavonoids, lipids, organic acids, terpenoids. Furthermore, correlation analysis indicated that these DEMs were significantly correlated with some specific bacteria, thereby helping in coordinating the root-zone soil community during conversion, while these bacteria were also correlated with soil properties.

**Conclusion:**

Overall, this study highlights the importance of bacterial communities during conversion of non-grain cultivated land to paddy field, which provided a scientific basis and supporting evidence for the renovation of non-grain cultivated land.

## 1 Background

Cultivated land is the major vehicle of food production, and demonstrates a central role in guaranteeing national food security, social stability and sustainable development (Lai et al., 2020; Lu et al., 2024). As the most populous country in the world, China feeds about 20% of the world population with just 7% of the world farmland (Cui and Shoemaker, 2018), thus China’s bumper grain plays a central role in maintaining global food security (Wu et al., 2023). However, due to the acceleration of urbanization, continuous upgrading of national food consumption demand from grain to pluralism, society’s blind pursuit of economic interests considering that comparative economic benefit gap between grain crops and economic forest fruits, and agricultural industrial policies, a significant tendency of using cultivated lands for non-grain production (such as bamboo shoots, perennial fruits, tea, vegetables, flowers) has widely occurred in recent years, in turn severely restricting local grain productivity, and leading to decline of cultivated land quantity and quality (Su et al., 2014; Yuan et al., 2019; Boulanger et al., 2022; Zhu et al., 2022; Chen et al., 2023; Deng et al., 2024).

To solve above-mentioned problems, the Chinese government has issued the “Opinions on Preventing the Non-Grain Production on Cultivated Land and Stabilizing Grain Production” on November 17, 2020, clearly understanding the profound urgency of preventing non-grain production on cultivated land and stabilizing grain production, earnestly grasping the national food security initiative, and establishing the reporting system of non-grain production on cultivated land so as to keep it strictly controlled (State Council of China, 2020). Since then, the Delta area of China has been actively carrying out conversion of non-grain cultivated land to paddy field, including consolidating non-grain paddy fields and restoring grain production functions to ensure food production, and improving the quality of cultivated land, which has significantly increased the scale of grain cultivation and thereby partly resolved issues caused by non-grainization (Shi et al., 2018; Liang and Geng, 2023). Therefore, it is of great significant to further solving non-grain related issues during the conversation of non-grain cultivated land to paddy field.

Over the past few decades, scholars have conducted lots of investigations from different perspectives on land use changes (Yu and Zhang, 2019; Meng et al., 2022; Hao et al., 2024). For example, different types of land use (agricultural, industrial, recreational, coastal, and residential areas) influenced soil physiochemical properties, the abundance of nitrifying bacteria, and microbial interactions in tropical urban soil (Das, 2023; Medriano et al., 2023). Land-use change from natural grasslands to shrub plantations, tree plantations, and arable lands altered patterns of soil biodiversity in arid lands of northwestern China (Li et al., 2018). The changes of typical six land-use types (forest, open forest, shrub, grassland, corn field and abandoned farmland) significantly affected soil phytolith-occluded organic carbon accumulation in Southwest China (Wang et al., 2023b). Conversion of upland crop to paddy field significantly changed soil water moisture and organic carbon contents, with increased bacterial diversity and changed bacterial community composition (Sun et al., 2021). Land use changes have been reported to be associated with the growth of different plant species, which shift the soil physiochemical properties and microbial community (Li et al., 2007; Jiang et al., 2016; Legrand et al., 2018; Lin et al., 2019; Sun et al., 2021). Farmland ecosystems exhibit distinct microclimates shaped by crops and land use changes by human intervention, which profoundly influence the composition and function of soil microbes (Deng et al., 2022; Agyekum et al., 2023).

Soil bacteria, the most prevalent microbes in soil, are vital for maintaining soil fertility and crop production by driving lots of soil ecosystem functions (including organic matter decomposition, humus formation, nutrients transformation, and suppression of soil-borne disease) (Ahmed et al., 2023; Chen et al., 2024). Simultaneously, soil nutrition gives rise to a vast diversity of soil bacteria (Wang X. et al., 2024), while soil metabolites strongly affect bacterial community structure and function (Bi et al., 2022). Obviously, these studies clearly revealed that soil bacteria exhibited important theoretical and practical implications for sustainable agricultural development by improving physiochemical properties and metabolites of soil. However, little attention has been paid on role of soil bacterial community structure and their ecological function during conversation of different types of non-grain production lands to paddy fields.

The objective of this study was to evaluate the impact of conversion of land use from non-grain cultivated land to paddy field on soil bacterial community structure, metabolite, and physicochemical properties by collecting soil samples from six types of non-grain cultivated land and the corresponding converted paddy fields, which will provide a scientific basis for the renovation of non-grain cultivated land, thus ensuring food security.

## 2 Methods

### 2.1 Soil sampling

On November 15, 2023, 72 soil samples (5–20 cm depth) were collected according to the method of (Butler et al., 2003) by mixing a total of nine random soil cores, which were picked up at the drip line around the crown of crops (including non-grain plants, and rice planted in the surrounding paddy fields converted from the corresponding non-grain lands), located in Jiande (loquat), Chun’an (mulberry), Tonglu (blueberry plant), Fuyang (grapevine), Lin’an (bamboo tree), and Yuhang (nursery stock), Hangzhou city (experiencing a subtropical monsoon climate with an average annual temperature of 17.8°C and precipitation of 1,454 mm), Zhejiang province, China (Table 1). Each treatment had six replicates. After passing through a 2 mm sieve, individual sample was partitioned into three: (a) air-dried at room temperature and passed through a 0.45 mm gauze for analysis of soil pH, soil organic matter (SOM), alkaline hydrolysis nitrogen (AHN), available phosphorus (AP), and available potassium (AK), (b) stored at 4°C for microbial biomass carbon (MBC) analysis, and (c) stored at −70°C for genome sequencing and metabolomic profiling analyses.

**Table 1:** The information of soil samples used in this study.

Treatments		Modes	Sites
JD-L	Loquat garden	Loquat garden to paddy field	Jiande
JD-L-R	Conversion of loquat garden to paddy field		
CA-M	Mulberry field	Mulberry field to paddy field	Chuan’an
CA-M-R	Conversion of mulberry field to paddy field		
TL-B	Blueberry garden	Blueberry garden to paddy field	Tonglu
TL-B-R	Conversion of blueberry garden to paddy field		
FY-G	Vineyard	Vineyard to paddy fields	Fuyang
FY-G-R	Conversion of vineyard to paddy fields		
LA-B	Bamboo garden	Bamboo gardens to paddy field	Lin’an
LA-B-R	Conversion of bamboo gardens to paddy field		
YH-S	Nursery stock base	Nursery stock base to paddy field	Yuhang
YH-S-R	Conversion of nursery stock base to paddy field		

### 2.2 Analyses of soil characteristics and MBC

Air-dried soil samples were used for analysis of soil characteristics. In detail, soil pH was estimated within 1: 5 soil suspension (soil: water, w/v) via pH meter (FE28, Met tlerToledo, Zurich, Switzerland) (Rathje, 1959). SOM was determined using K_2_Cr_2_O_7_ oxidation heating method (Nelson and Sommers, 1996). AHN was measured by conductometric titration (Chen et al., 2016). AP and AK were extracted with ammonium lactate solution and then analyzed using spectrophotometry and flame photometry, respectively (Tian et al., 2021). In contrast, fresh soil samples were used to determine MBC, which was performed by employing chloroform fumigation-extraction method (Vance et al., 1987).

### 2.3 Soil genome sequencing

Following the extraction of DNAs using the E.Z.N.ATM Mag-Bind Soil DNA Kit (OMEGA, USA), soil bacterial diversity were determined by amplifing the V3–V4 region of the bacterial 16S rRNA gene using two universal primers 341F (5′-CCTACGGGNGGCWGCAG-3′) and 805R (5′-GACTACHVGGGTATCTAATCC-3′) (Wu et al., 2015), which was carried out as described by Li et al. (2024). The reaction mixture of PCR contained 2 × Hieff® Robust PCR Master Mix (15 μL), 10 μM primer 341F (1 μL), 10 μM primer 805R (1 μL), ddH_2_O (12 μL), and DNA (1 μL). PCR was run at 95°C for 3 min; 95°C for 30 s, 45°C for 30 s, 72°C for 30 s, 5 cycles; 95°C for 30 s, 55°C for 30 s, 72°C for 30 s, 20 cycles; 72°C for 5 min. The final amplicon was detected by 2% agarose gel, purified by Hieff NGS™ DNA selection beads (Yeasen, China), quantified using a Qubit 4.0 (Thermo, USA), and subsequently pair-end (2 × 250 bp) sequenced on an Illumina MiSeq platform (Sangon BioTech, Shanghai, China).

After the sequencing process, the primers were cut off using Cutadapt (v3.5) (Martin, 2011). The short Illumina reads were assembled adopting PEAR (v0.9.8), and then the reads with Phred33 score of less than 20 were removed via Trimmomatic (v0.39) to ensure data integrity (Bolger et al., 2014). After, raw reads were further filtered, denoised, and concatenated by DADA2 (v1.14.0) (Callahan et al., 2016), the chimera was then clustered into operational taxonomic units (OTUs) using Usearch (v11.0.667) with a 97% similarity cutoff. After selection of the representative read of each cluster using QIIME (v2020.06), taxonomic classification of each OTU was performed with Silva (v138.1) using the RDP classifier (v2.12) (Wang et al., 2007; Quast et al., 2012; Wang et al., 2023a).

### 2.4 Soil metabolomics assay

Metabolic assay was carried out as described by Li et al. (2024) using liquid chromatography-mass spectrometry (LC–MS) system (Vanquish, Thermo), in which LC coupled to an Orbitrap Exploris 120 mass spectrometer (Orbitrap MS, Thermo). To assess the quality and reproducibility of data, pooled quality control samples were included by adding equal amount of all sample supernatants. The original data obtained via LC–MS was changed into mzXML format by ProteoWizard. Peak extraction, peak alignment, and time retention correction were, respectively, performed by XCMS. The peak area was corrected by SVR, and the peaks with detection rate lower than 50% in each group of samples were discarded. Afterwards, metabolite annotation was executed against an in-house MS2 database (Sangon BioTech, Shanghai, China).

### 2.5 Statistical analysis

The comparative analysis were performed on each land type between the non-grain cultivated lands and the corresponding converted paddy fields, while the conversation of each land type from non-grain cultivated land to paddy field was arranged in a county, ensuring consistency and minimize variability across sampling locations. One-way analysis of variance (ANOVA) tests were adopted to analyze variance using SPSS (v16.0) (Chicago, USA). To assess bacterial abundance and α-diversity, the OTU richness and α-diversity indexes (including Chao1, Shannon, and Simpson indexes) were visualized via Origin (v2022) (Hampton, USA) after normalizing data by Usearch (v11) (California, USA). To assess changes in the bacterial community structure, principal component analysis (PCA) was performed using Bray–Curtis dissimilarity matrix (Ramette, 2007). The significant differences between groups were tested by permutational multivariate ANOVA (PERMANOVA), with 999 permutations used to calculate *p*-values (Dixon, 2003). In visualizing bacterial community composition, relative abundances (RAs) and heat maps of dominant bacteria taxa were conducted using Origin (v2022). To evaluate the influence of biomarkers on different groups, linear discriminant analysis (LDA) effect size (LEfSe) was carried out using LEfSe Galaxy based on the LDA score (Segata et al., 2011). To investigate how the conversion processes affect soil bacterial co-occurrence patterns, co-occurrence networks were constructed using a SparCC correlation matrix, based on RAs (>1%) and statistically significant correlations of RAs (*p* < 0.01, SparCC’s coefficient *N* > 0.22 or < −0.22) among OTUs, and visualized via Gephi (v0.9.2) (Friedman and Alm, 2012; Weiss et al., 2016; Gloor et al., 2017; Peschel et al., 2021; Yu et al., 2022). To explore the differences in metabolites under different groups, orthogonal projections to latent structures discriminant analysis (OPLS-DA), volcano plots, pathway enrichment analysis of differential metabolites were adopted with the MetaboAnalyst 4.0 platform. To gain a better understanding of the potential association between differentially expressed metabolites (DEMs) and bacteria, the correlation heat maps were clustered as described by Hollander et al. (2015) based on the Spearman’s rank correlation coefficient among the top 20 RA of root-zone soil bacteria and significant DEMs with largest variable importance in projection (VIP). Meanwhile, redundancy discriminant analysis (RDA) was performed to investigate the impact of different environmental factors (such as pH and nutrition) on microbial community structure by Origin (v2022, Hampton, MA, USA).

## 3 Results

### 3.1 Impacts on soil pH and chemical properties

**Figure 1:**
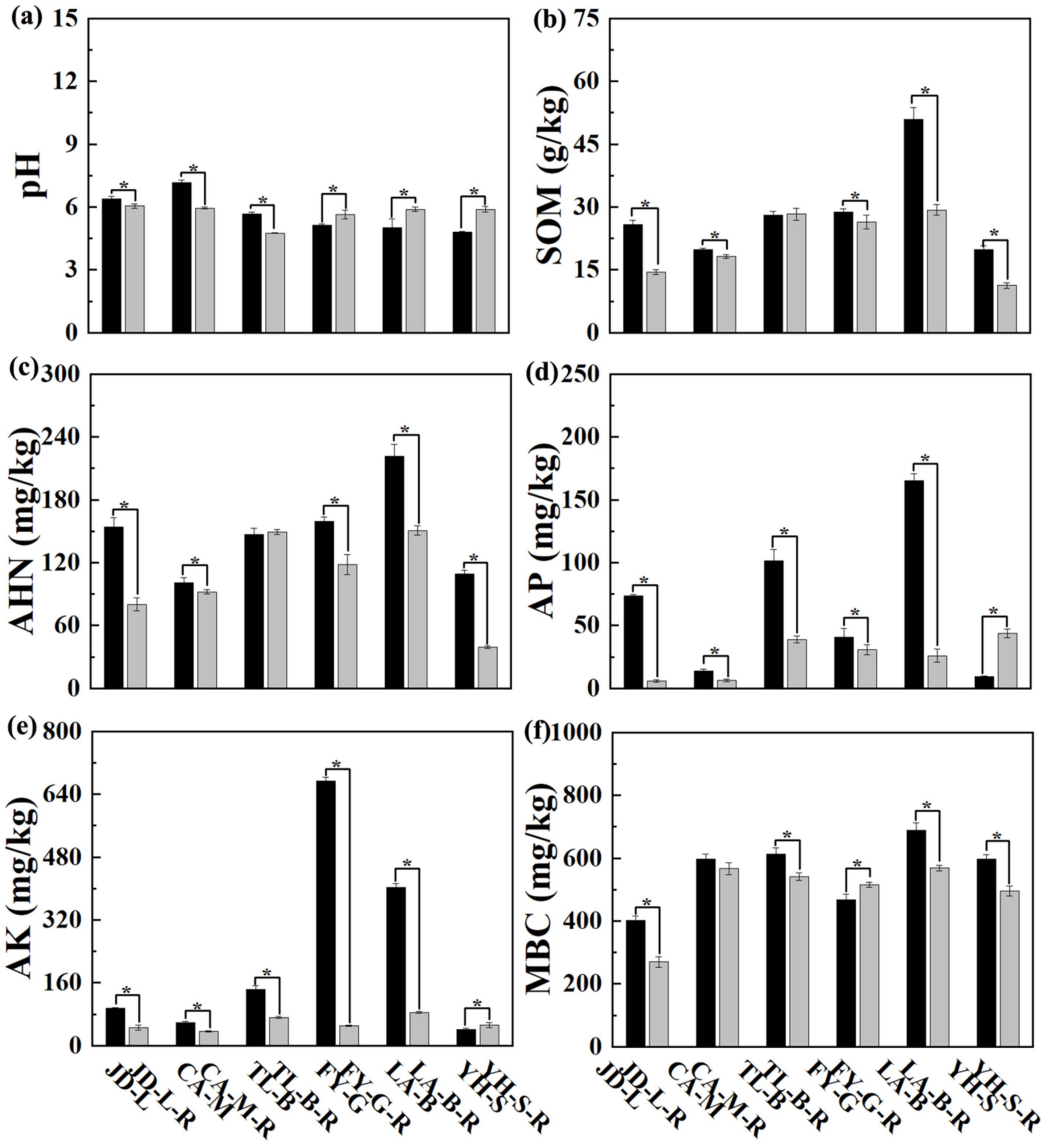
The pH and chemical properties of the root-zone soil between non-grain cultivated land and paddy field under different groups.

Results from this study showed that soil physicochemical properties were differentially affected by conversion of six non-grain cultivated lands to paddy field, while the effect depends on both the type of non-grain cultivated lands and the kind of soil parameters. Indeed, the soil pH was significantly (*p* < 0.05) reduced by conversion of loquat garden, mulberry field, blueberry garden to paddy field (5.30–16.97%), but increased by conversion of vineyard, bamboo garden and nursery stock base to paddy field (10.04–22.62%). However, conversion of six non-grain cultivated lands to paddy field significantly (*p* < 0.05) decreased the SOM (8.08–43.85%), AHN (8.90–64.14%), AP (24.98–91.97%), AK (38.74–92.52%), and MBC (5.12–32.99%), except a slight increase in the SOM (0.96%) and AHN (1.63%) by conversion of blueberry garden to paddy field, a significant (*p* < 0.05) increase in the AP (372.11%) and AK (25.50%) by conversion of nursery stock base to paddy field, and in the MBC (10.20%) by conversion of vineyard to paddy field (Figure 1; Table 2).

**Table 2:** Soil properties as affected by conversation of non-grain cultivated land to paddy field.

Treatments	pH	SOM (g/kg)	AHN (mg/kg)	AP (mg/kg)	AK (mg/kg)	MBC (mg/kg)
JD-L	6.40 ± 0.11	25.77 ± 1.12	153.91 ± 4.26	73.39 ± 1.28	94.47 ± 2.87	402.60 ± 14.01
JD-L-R	6.06 ± 0.10 *	14.47 ± 0.59 *	80.03 ± 4.51 *	5.90 ± 0.81 *	46.26 ± 2.07 *	269.78 ± 16.44 *
CA-M	7.17 ± 0.10	19.80 ± 0.28	101.10 ± 4.79	13.70 ± 1.55	59.26 ± 2.27	596.95 ± 16.22
CA-M-R	5.95 ± 0.04 *	18.20 ± 0.51 *	92.10 ± 2.29 *	6.16 ± 0.98 *	36.30 ± 2.23 *	566.41 ± 18.32
TL-B	5.66 ± 0.10	28.02 ± 0.91	147.10 ± 5.85	101.60 ± 5.99	142.46 ± 10.07	613.31 ± 18.68
TL-B-R	4.76 ± 0.01 *	28.28 ± 1.40	149.50 ± 2.39	38.84 ± 2.70 *	71.90 ± 2.89 *	541.66 ± 11.40 *
FY-G	5.13 ± 0.07	28.77 ± 0.78	159.56 ± 4.25	40.89 ± 2.08	673.05 ± 10.85	468.00 ± 16.91
FY-G-R	**5.65 ± 0.22 #**	26.42 ± 1.66	118.14 ± 6.92 *	30.67 ± 1.99 *	50.31 ± 1.74 *	**515.74 ± 8.10 #**
LA-B	5.00 ± 0.42	50.98 ± 2.81	221.72 ± 7.11	165.10 ± 5.31	402.82 ± 9.11	688.84 ± 23.97
LA-B-R	**5.89 ± 0.10 #**	29.30 ± 1.31 *	150.68 ± 4.54 *	25.91 ± 1.79 *	84.49 ± 2.80 *	568.26 ± 9.89 *
YH-S	4.81 ± 0.03	19.76 ± 0.93	109.44 ± 3.16	9.26 ± 0.54	41.14 ± 3.13	596.16 ± 15.16
YH-S-R	**5.89 ± 0.13 #**	11.27 ± 0.68 *	39.24 ± 1.30 *	**43.73 ± 3.36 #**	**51.64 ± 0.76 #**	496.37 ± 15.97 *

* and # indicate statistically significant increases or decreases, respectively, compared to corresponding non-grain cultivated land (*p* < 0.05). JD-L, loquat garden; JD-L-R, conversion of loquat garden to paddy field; CA-M, mulberry field; CA-M-R, conversion of mulberry field to paddy field; TL-B, blueberry garden; TL-B-R, conversion of blueberry garden to paddy field; FY-G, vineyard; FY-G-R, conversion of vineyard to paddy field; LA-B, bamboo garden; LA-B-R, conversion of bamboo garden to paddy field; YH-S, nursery stock base; YH-S-R, conversion of nursery stock base to paddy field. SOM, soil organic matter; AHN, alkaline hydrolysis nitrogen; AP, available phosphorus; AK, available potassium; MBC, microbial biomass carbon.Values significantly increased are shown in bold.

### 3.2 Impacts on soil bacterial community characteristics

#### 3.2.1 Soil bacterial community diversity

**Figure 2:**
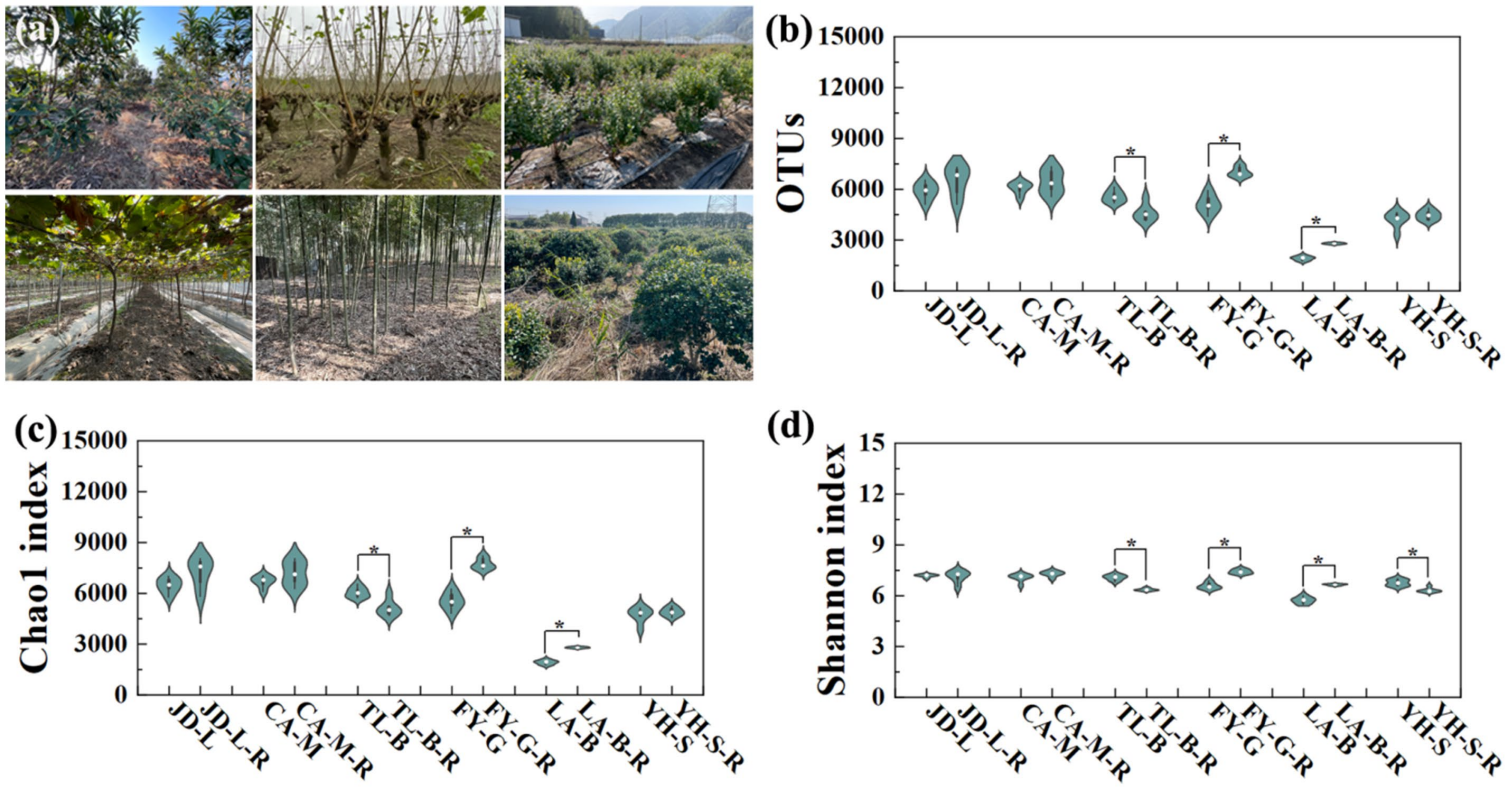
Six diverse conversion modes from non-grain cultivated lands to paddy field **(a)**, and the bacterial OTU distribution **(b)**, Chao1 **(c)**, and Shannon **(d)** index under different groups. “*” above columns indicate statistical significant differences (*p* < 0.05). A total of 363,752 OTUs (5,079–7,294 for Jiande, 5,454–7,333 for Chun’an, 4,138–6,135 for Tonglu, 4,399–7,486 for Fuyang, 1,750–2,853 for Lin’an, and 3,339–4,899 for Yuhang) from 21 bacterial phyla were identified.

In six diverse conversion modes from non-grain cultivated lands to paddy field (Figure 2a), a total of 363,752 OTUs from 21 bacterial phyla were identified, while the distribution of OTUs across all treatments was shown in Figure 2b. Generally, the conversion of non-grain cultivated land to paddy field significantly changed the richness and diversity of bacteria in the root-zone soils. In detail, the number of bacterial OTUs in the converted paddy fields was increased by 5.67–45.26% except a significant 17.64% reduction by conversion of blueberry garden to paddy field. The α-diversity analysis was chosen to evaluate the bacterial community (Figures 2c,d), while the trend of bacterial Chao1 index was basically same as OTUs, with increases of 3.62–45.22% in the converted paddy fields, and a significant reduction of 15.48% by conversion of blueberry garden to paddy field. Whereas, the Shannon index was increased by 3.17–16.27% by conversion of mulberry field, vineyard and bamboo garden to paddy fields, but decreased by 0.82–10.11% by conversion of the other three non-grain cultivated land to paddy field.

**Figure 3:**
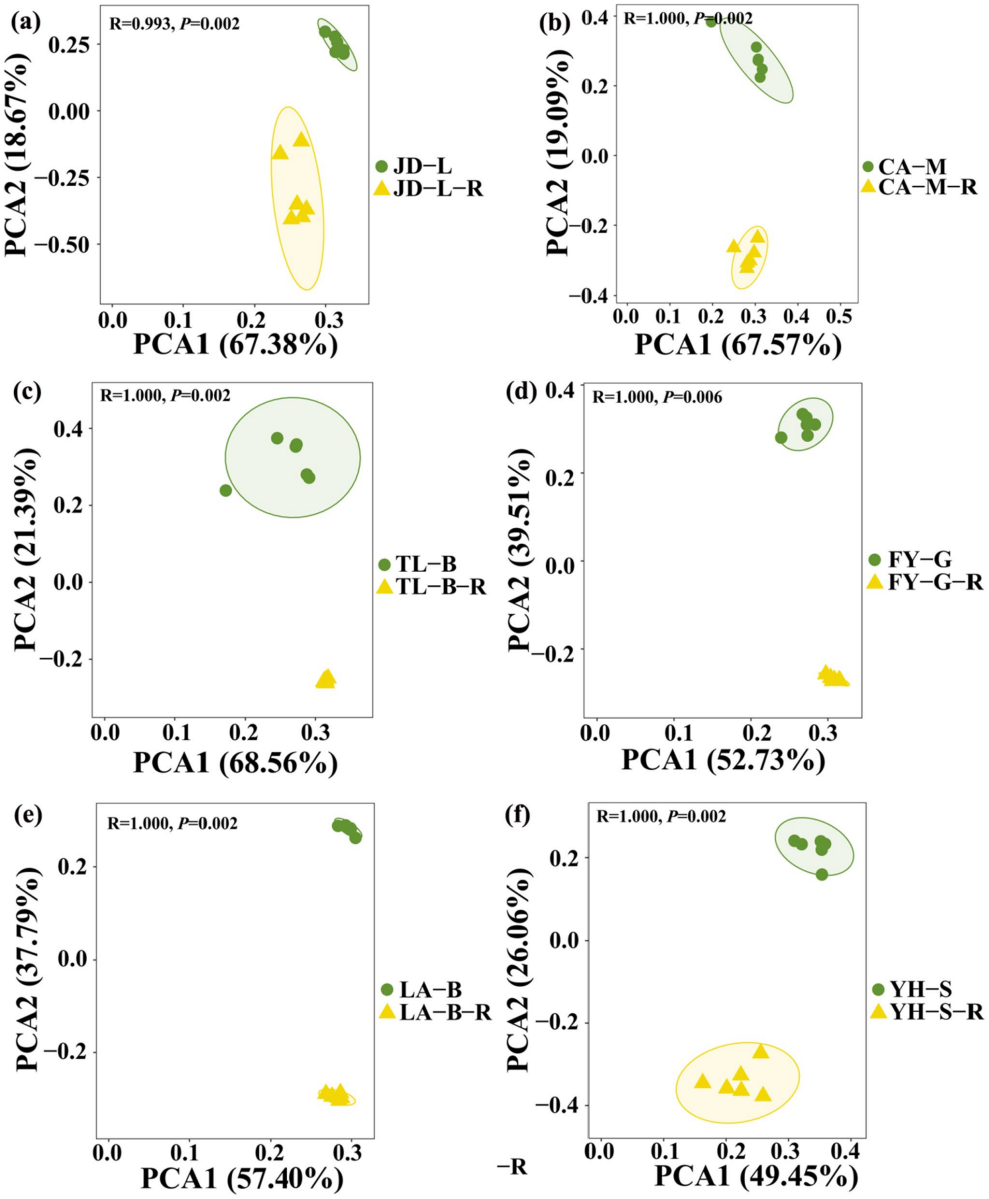
Principal component analysis (PCA) of the soil root-zone bacterial communities at the OTU level under different groups. The conversion of loquat garden to paddy field **(a)**, conversion of mulberry field to paddy field **(b)**, conversion of blueberry garden to paddy field **(c)**, conversion of vineyard to paddy field **(d)**, conversion of bamboo garden to paddy field **(e)**, and conversion of nursery stock base to paddy field **(f)**. Ellipses have been drawn for each treatment with a confidence limit of 0.95.

To further examine the effect of conversion of non-grain cultivated land to paddy field on root-zone soil bacterial communities, the PCA analysis at the OTU level was carried out based on the Bray-Curtis distance (Figure 3). Results from this study indicated that the conversion of non-grain cultivated land to paddy field significantly changed the bacterial community structure of the root-zone soil, while the effect depends on the type of conversion. Indeed, the root-zone soil bacterial communities of paddy fields converted from six non-grain cultivated lands formed two significantly different groups, and all groups were well separated from each other. Furthermore, the PCA1 and PCA2 revealed 49.45–68.56% and 18.67–39.51% of the variability in the bacterial communities, respectively, while the results of PERMANOVA indicated that the type of non-grain cultivated land explained 99.3–100% of the variation (*p* = 0.002–0.006).

#### 3.2.2 Soil bacterial community structure

**Figure 4:**
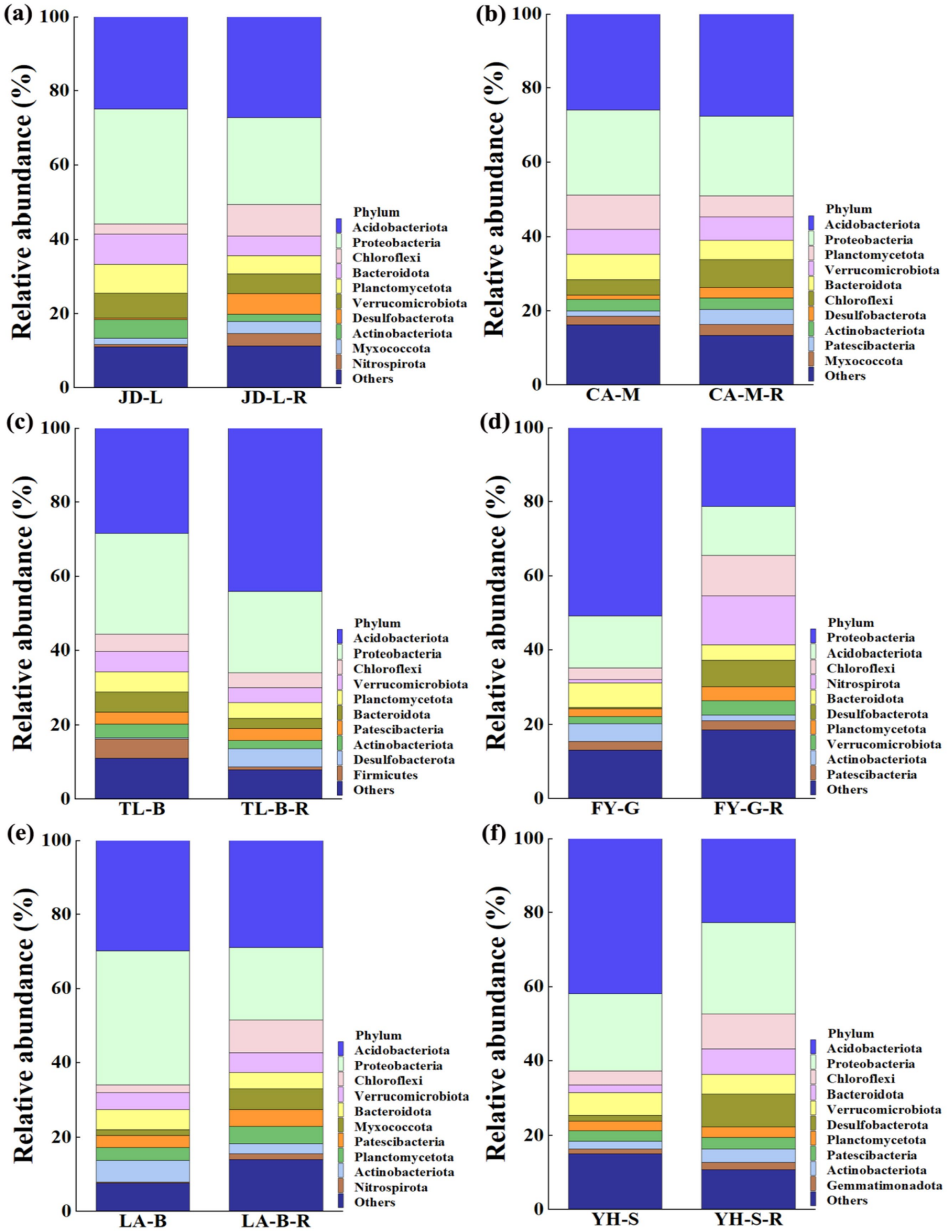
Relative abundance (RA) of the top 10 dominant bacteria at the phlyum level under different groups. The conversion of loquat garden to paddy field **(a)**, conversion of mulberry field to paddy field **(b)**, conversion of blueberry garden to paddy field **(c)**, conversion of vineyard to paddy field **(d)**, conversion of bamboo garden to paddy field **(e)**, and conversion of nursery stock base to paddy field **(f)**.

Results indicated that conversion of non-grain cultivated land to paddy field led to significant changes in the bacterial community composition of the root-zone soil at the phylum level, while the relative abundance of the top 10 bacterial phyla was noted across all soil samples (Figure 4). In details, conversion of loquat garden to paddy field significantly increased Desulfobacterota (12.43 fold), Nitrospirota (4.29 fold), Chloroflexi (1.97 fold), and Myxococcota (0.92 fold), but significantly decreased Actinobacteriota (0.62 fold) (Figure 4a). Conversion of mulberry field to paddy field significantly increased Patescibacteria (1.84 fold), Desulfobacterota (1.26 fold), and Chloroflexi (0.81 fold) (Figure 4b). Conversion of blueberry garden to paddy field significantly increased Desulfobacterota (15.00 fold) and Acidobacteriota (0.55 fold), but significantly decreased Firmicutes (0.85 fold) and Bacteroidota (0.50 fold) (Figure 4c). Conversion of vineyard to paddy field significantly increased Desulfobacterota (21.50 fold), Nitrospirota (14.54 fold), Chloroflexi (2.41 fold), Verrucomicrobiota (0.97 fold) and Planctomycetota (0.82 fold), but significantly decreased Actinobacteriota (0.68 fold) and Proteobacteria (0.58 fold) (Figure 4d). Conversion of bamboo garden to paddy field significantly increased Nitrospirota (7.53 fold), Chloroflexi (3.08 fold), and Myxococcota (2.55 fold), but significantly decreased Actinobacteriota (0.53 fold) (Figure 4e). Conversion of nursery stock base to paddy field significantly increased Desulfobacterota (4.33 fold), Bacteroidota (2.35 fold), Chloroflexi (1.48 fold), and Actinobacteriota (0.69 fold) (Figure 4f).

**Figure 5:**
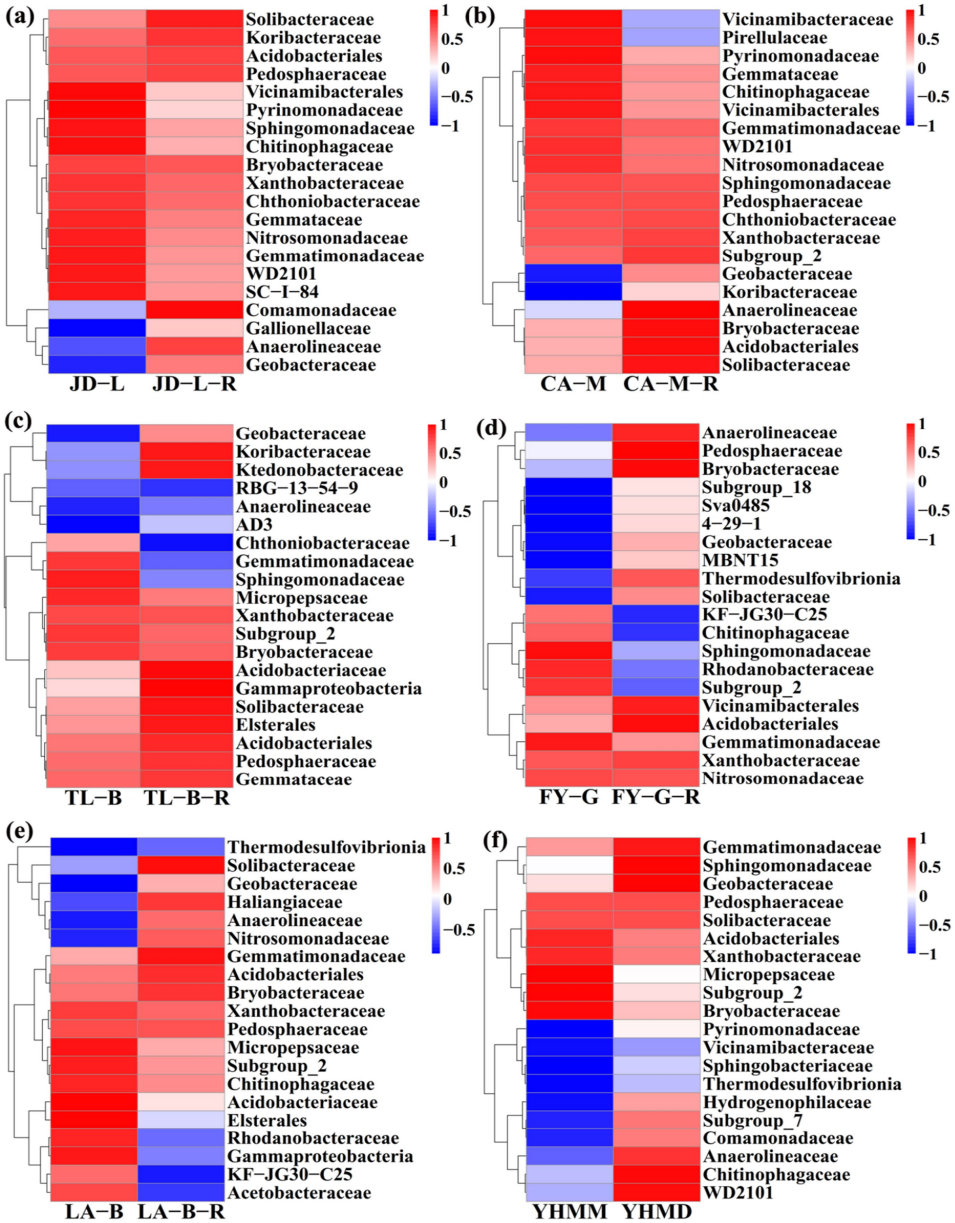
Heat map at the family level. The conversion of loquat garden to paddy field **(a)**, conversion of mulberry field to paddy field **(b)**, conversion of blueberry garden to paddy field **(c)**, conversion of vineyard to paddy field **(d)**, conversion of bamboo garden to paddy field **(e)**, and conversion of nursery stock base to paddy field **(f)**. The tree plot represents a clustering analysis of the top 20 bacteria at the family level according to their Person correlation coefficient matrix and relative abundance.

Furthermore, the difference in relative abundance composition of soil bacterial community among all treatments at the family level was further visually illustrated through heat maps (Figure 5). In detail, the converted paddy fields were enriched with Anaerolineaceae, Bryobacteraceae, Comamonadaceae, Gallionellaceae, Geobacteraceae, Haliangiaceae, Hydrogenophilaceae, Koribacteraceae, Ktedonobacteraceae, MBNT15, Nitrosomonadaceae, Pedosphaeraceae, Solibacteraceae, Subgroup_7, Subgroup_18, Sva0485, 4–29-1, Thermodesulfovibrionia, and WD2101, but were reduced with Acetobacteraceae, Chthoniobacteraceae, Elsterales, Gammaproteobacteria, Gemmatimonadaceae, KF-JG30-C25, Pirellulaceae, Rhodanobacteraceae, Sphingomonadaceae, Subgroup_2, and Vicinamibacteraceae. The results demonstrate that rice cultivation can increase or reduce the presence of certain species, accounting in a change in the root-zone soil bacterial community structure. Notably, these bacteria may have significant potential in colonizing and altering soil bacterial communities during conversion of non-grain cultivated land to paddy field.

#### 3.2.3 Soil microbiome and biomarker

**Figure 6:**
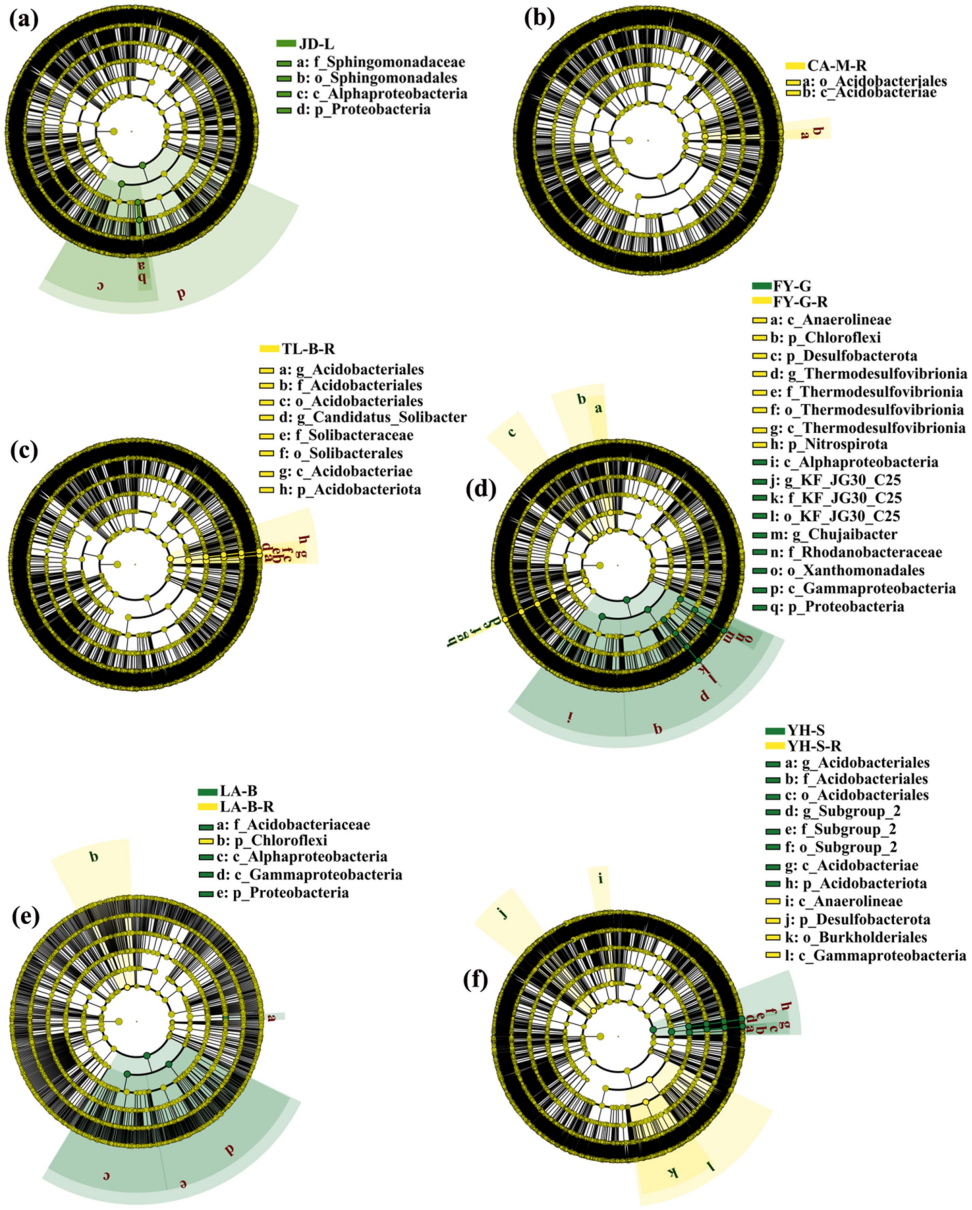
Linear discriminant analysis (LDA) effect size (LEfSe) of the root-zone soil bacterial taxa. The conversion of loquat garden to paddy field **(a)**, conversion of mulberry field to paddy field **(b)**, conversion of blueberry garden to paddy field **(c)**, conversion of vineyard to paddy field **(d)**, conversion of bamboo garden to paddy field **(e)**, and conversion of nursery stock base to paddy field **(f)**.

LEfSe was performed to identify the biomarkers with the most difference in the root-zone soil bacterial communities between non-grain cultivated land and the converted paddy fields (Figure 6). Results showed that a total of 48 bacteria biomarkers (LDA > 4.5, *p* < 0.05) were found in all groups. Indeed, the loquat garden was enriched with Alphaproteobacteria, Proteobacteria, Sphingomonadaceae, and Sphingomonadales; the paddy field converted from mulberry field was enriched with Acidobacteriales and Acidobacteriae; the paddy field converted from blueberry field was enriched with three types of *Acidobacteriales*, Acidobacteriae, Acidobacteriota, *Candidatus_Solibacter*, Solibacteraceae, and Solibacterales; the vineyard was enriched with Anaerolineae, Chloroflexi, Desulfobacterota, four types of *Thermodesulfovibrionia*, and Nitrospirota, while the converted paddy field was enriched with Alphaproteobacteria, *Chujaibacter*, Gammaproteobacteria, three types of *KF_JG30_C25*, Proteobacteria, Rhodanobacteraceae, and Xanthomonadales; the bamboo garden was enriched with Acidobacteriaceae, Alphaproteobacteria, Gammaproteobacteria, and Proteobacteria, while the converted paddy field was enriched with Chloroflexi; the nursery stock base was enriched with three types of *Acidobacteriales*, Acidobacteriae, Acidobacteriota, Anaerolineae, and three types of *Subgroup_2*, while the converted paddy field was enriched with Burkholderiales, Desulfobacterota, and Gammaproteobacteria. As stated above, 25 and 23 bacteria biomarkers were associated with six kinds of non-grain cultivated land and the converted paddy fields, respectively.

#### 3.2.4 Co-occurrence networks of root-zone soil bacteria

**Figure 7:**
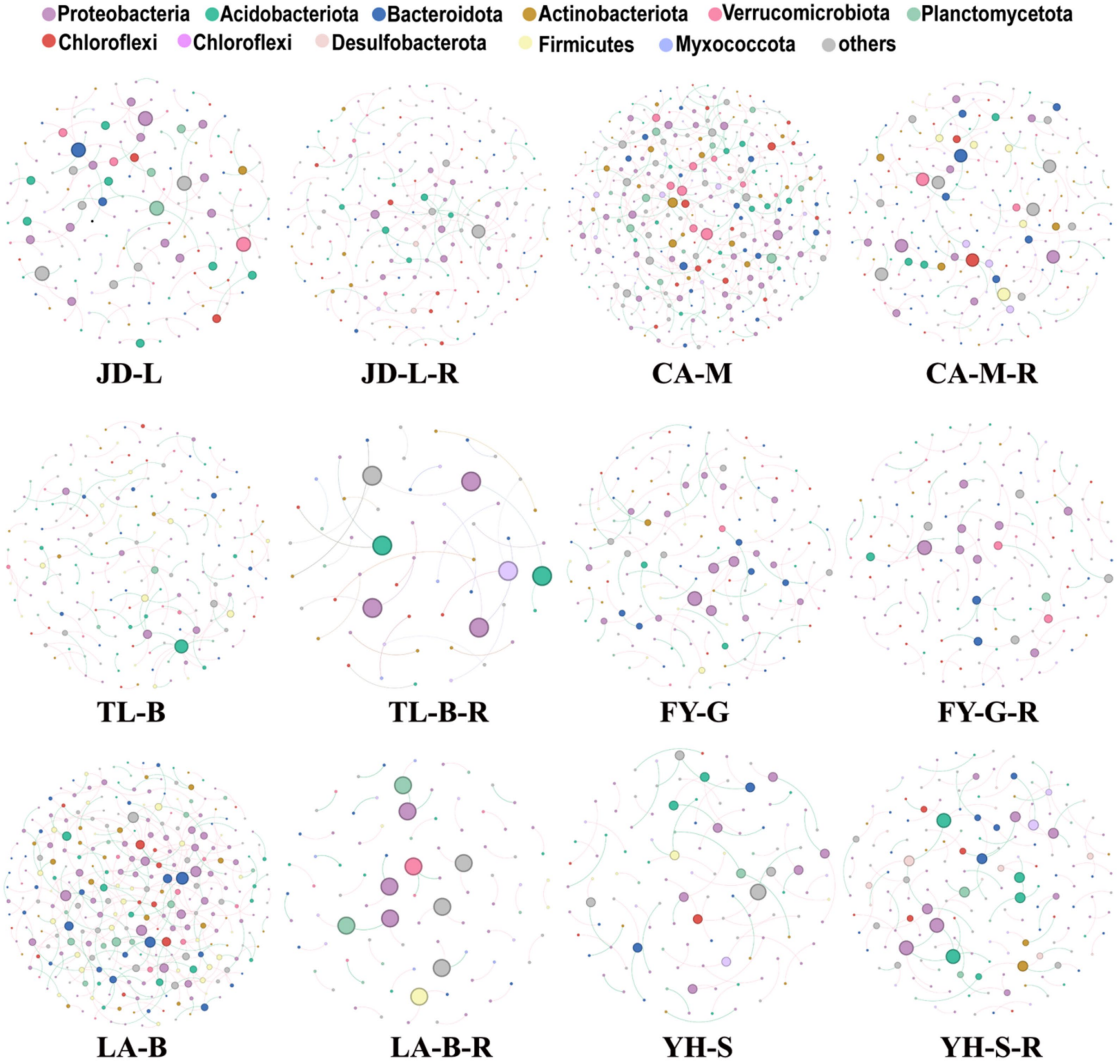
The differences on the co-occurrence patterns of soil bacterial communities. Networks were constructed at the OTU level. The size of the nodes (OTUs) represented the relative abundance (RA) of the bacteria, and the nodes were colored according to the phylum.

To visualize the complexity and stability of soil bacterial community responses to conversion of non-grain cultivated land to paddy field in each group, co-occurrence networks were built, and then the topological properties were estimated to characterize differences between different groups (Figure 7; Table 3). Nodes represent microbes derived from OTUs, edges (positive/negative links between nodes) correspond to potential associations between nodes, while modularity indicates the presence of dense cluster of related nodes embedded within the network. In other word, fewer nodes or edges signify a less interconnected community and higher modularity represent higher structural stability of network (Freundt, 2021; Ma et al., 2021; Yang et al., 2022). Results showed that the nodes, edges and average degree was decreased by 32.17, 54.46, and 32.86%, respectively, in the paddy fields converted from mulberry field, decreased by 71.64, 76.47, and 17.00%, respectively, in the paddy fields converted from blueberry garden, increased by 1.23% and decreased by 8.00 and 9.13%, respectively, in the paddy fields converted from vineyard, decreased by 69.82, 84.36, and 48.19%, respectively, in the paddy fields converted from bamboo garden, increased by 29.63, 45.10, and 11.99%, respectively, in the paddy fields converted from loquat garden, increased by 48.67, 68.18, and 13.10%, respectively, in the paddy fields converted from nursery stock base. Whereas conversion of non-grain cultivated land to paddy field resulted in 4.38–65.17% increase in the modularity, suggesting that the bacterial community structure became more stable in the converted paddy field.

**Table 3:** Key topological parameters of bacterial co-occurrence networks under different groups.

Network properties	Nodes	Edges	Average degree	Modularity
JD-L	162	102 (positive: 34, negative: 68)	1.259	−1.050
JD-L-R	210	148 (positive: 47, negative: 101)	1.410	−1.004
CA-M	345	314 (positive: 106, negative: 208)	1.820	−1.051
CA-M-R	234	143 (positive: 46, negative: 97)	1.222	−0.939
TL-B	201	136 (positive: 50, negative: 86)	1.353	−1.581
TL-B-R	57	32 (positive: 11, negative: 21)	1.123	−1.284
FY-G	163	100 (positive: 35, negative: 65)	1.227	−1.200
FY-G-R	165	92 (positive: 20, negative: 72)	1.115	−0.418
LA-B	285	307 (positive: 107, negative: 200)	2.154	−1.103
LA-B-R	86	48 (positive: 14, negative: 34)	1.116	−0.792
YH-S	113	66 (positive: 25, negative:41)	1.168	−1.897
YH-S-R	168	111 (positive: 42, negative: 69)	1.321	−1.593

### 3.3 Soil metabolomics

**Figure 8:**
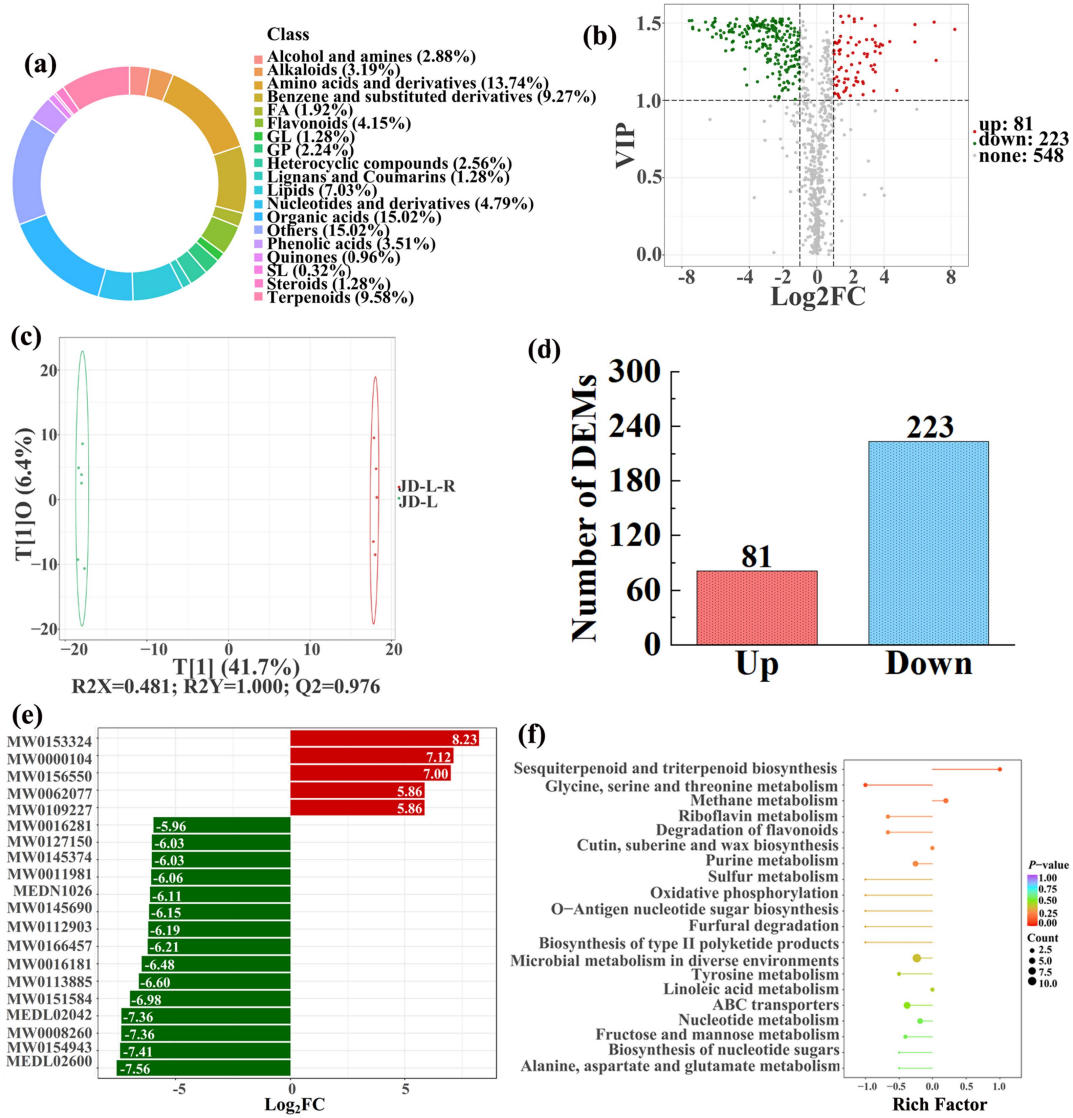
Donut plots showing metabolite classification and proportion **(a)**, volcano plot **(b)**, orthogonal projection to latent structures-discriminant analysis (OPLS-DA) score map **(c)**, the number of DEMs **(d)**, top 20 DEMs with largest VIP **(e)**, KEGG enrichment analysis of differential soil metabolites in conversion of loquat garden to paddy field (JD-L-R) *vs* loquat garden (JD-L) **(f)**.

**Figure 9:**
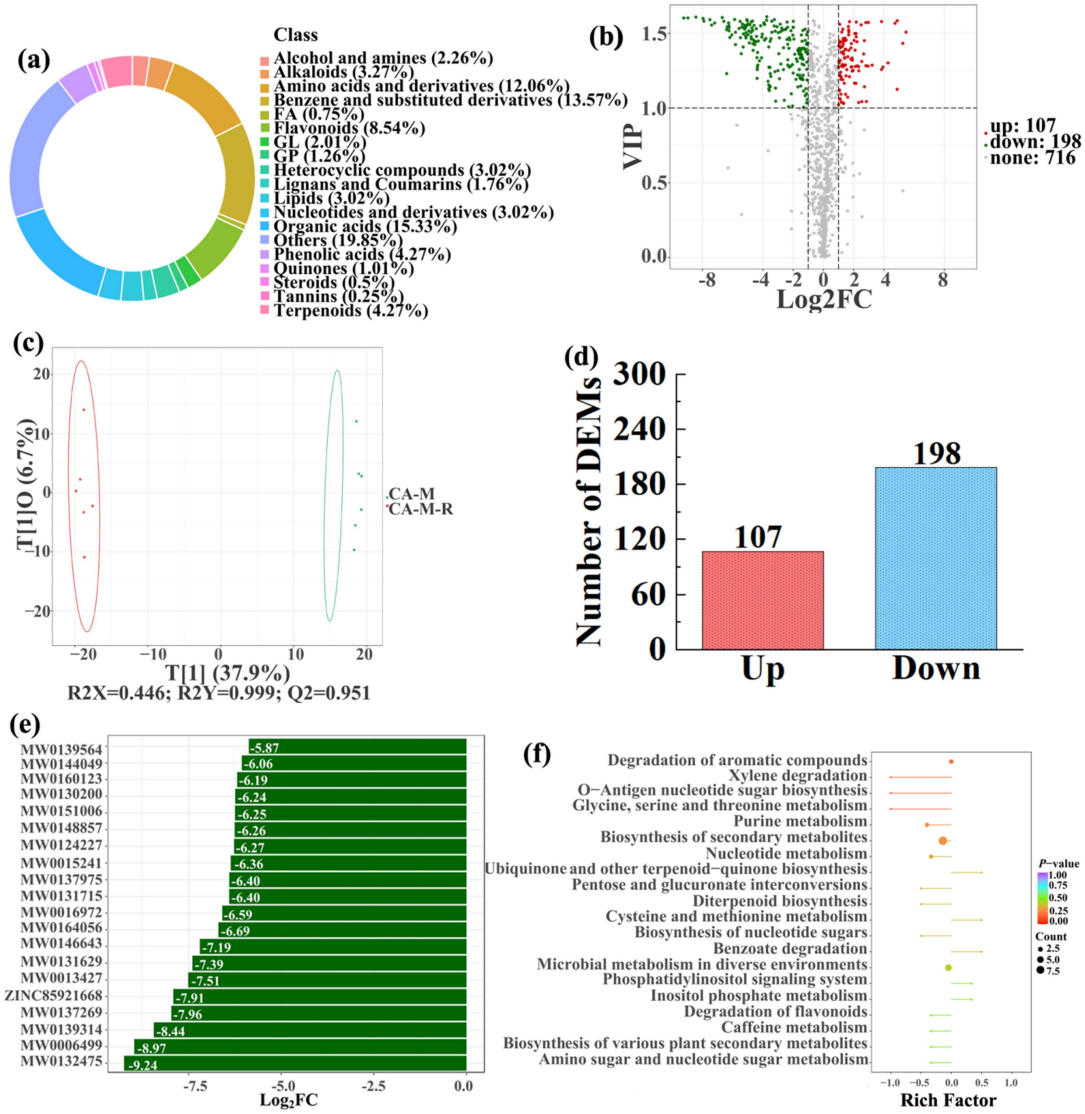
Donut plots showing metabolite classification and proportion **(a)**, volcano plot **(b)**, orthogonal projection to latent structures-discriminant analysis (OPLS-DA) score map **(c)**, the number of DEMs **(d)**, top 20 DEMs with largest VIP **(e)**, KEGG enrichment analysis of differential soil metabolites in conversion of mulberry field to paddy field (CA-M-R) *vs* mulberry field (CA-M) **(f)**.

**Figure 10:**
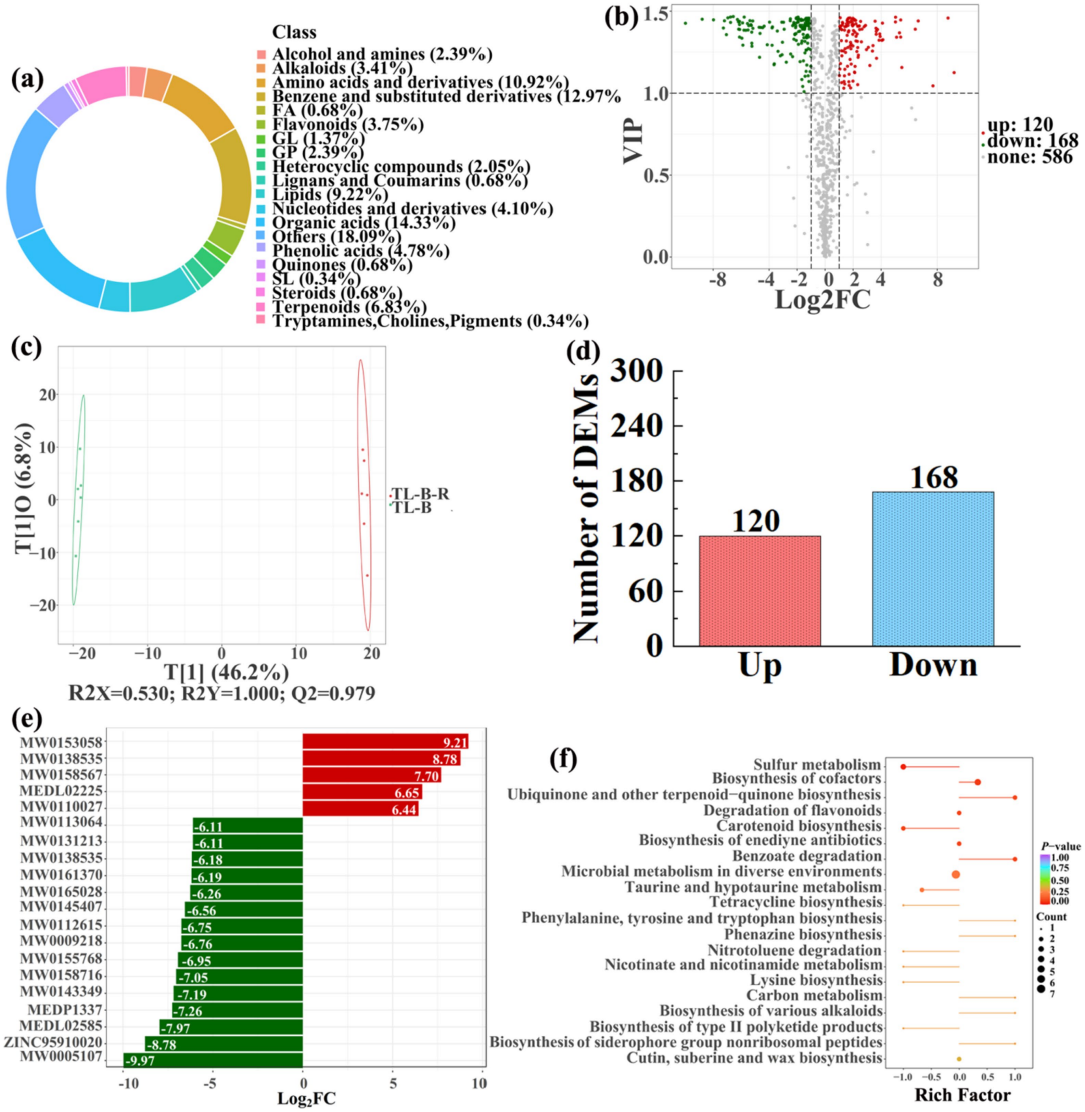
Donut plots showing metabolite classification and proportion **(a)**, volcano plot **(b)**, orthogonal projection to latent structures-discriminant analysis (OPLS-DA) score map **(c)**, the number of DEMs **(d)**, top 20 DEMs with largest VIP **(e)**, KEGG enrichment analysis of differential soil metabolites in conversion of blueberry garden to paddy field (TL-B-R) *vs* blueberry garden (TL-B) **(f)**.

**Figure 11:**
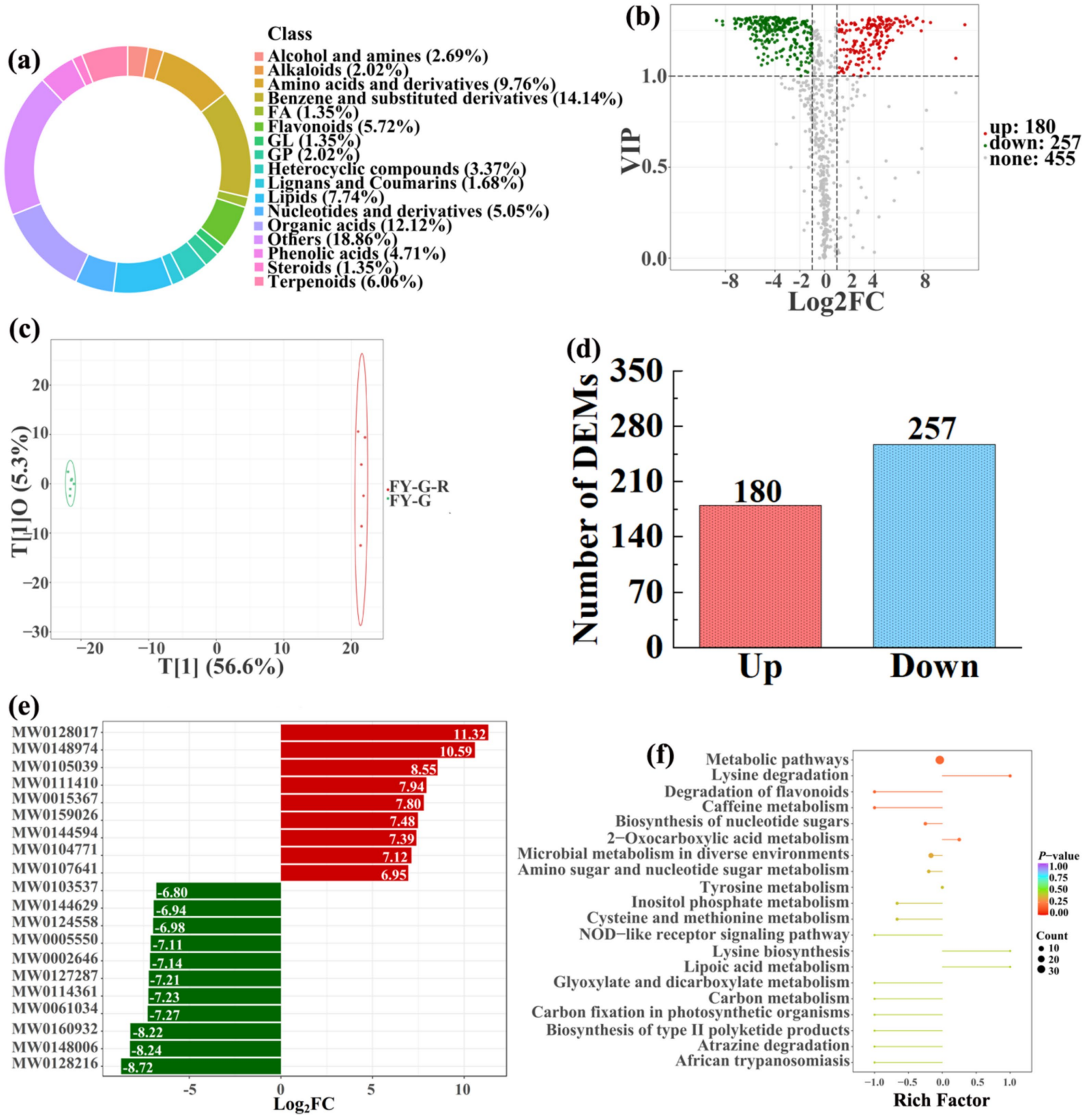
Donut plots showing metabolite classification and proportion **(a)**, volcano plot **(b)**, orthogonal projection to latent structures-discriminant analysis (OPLS-DA) score map **(c)**, the number of DEMs **(d)**, top 20 DEMs with largest VIP **(e)**, KEGG enrichment analysis of differential soil metabolites in conversion of vineyard to paddy field (FY-G-R) *vs* vineyard (FY-G) **(f)**.

**Figure 12:**
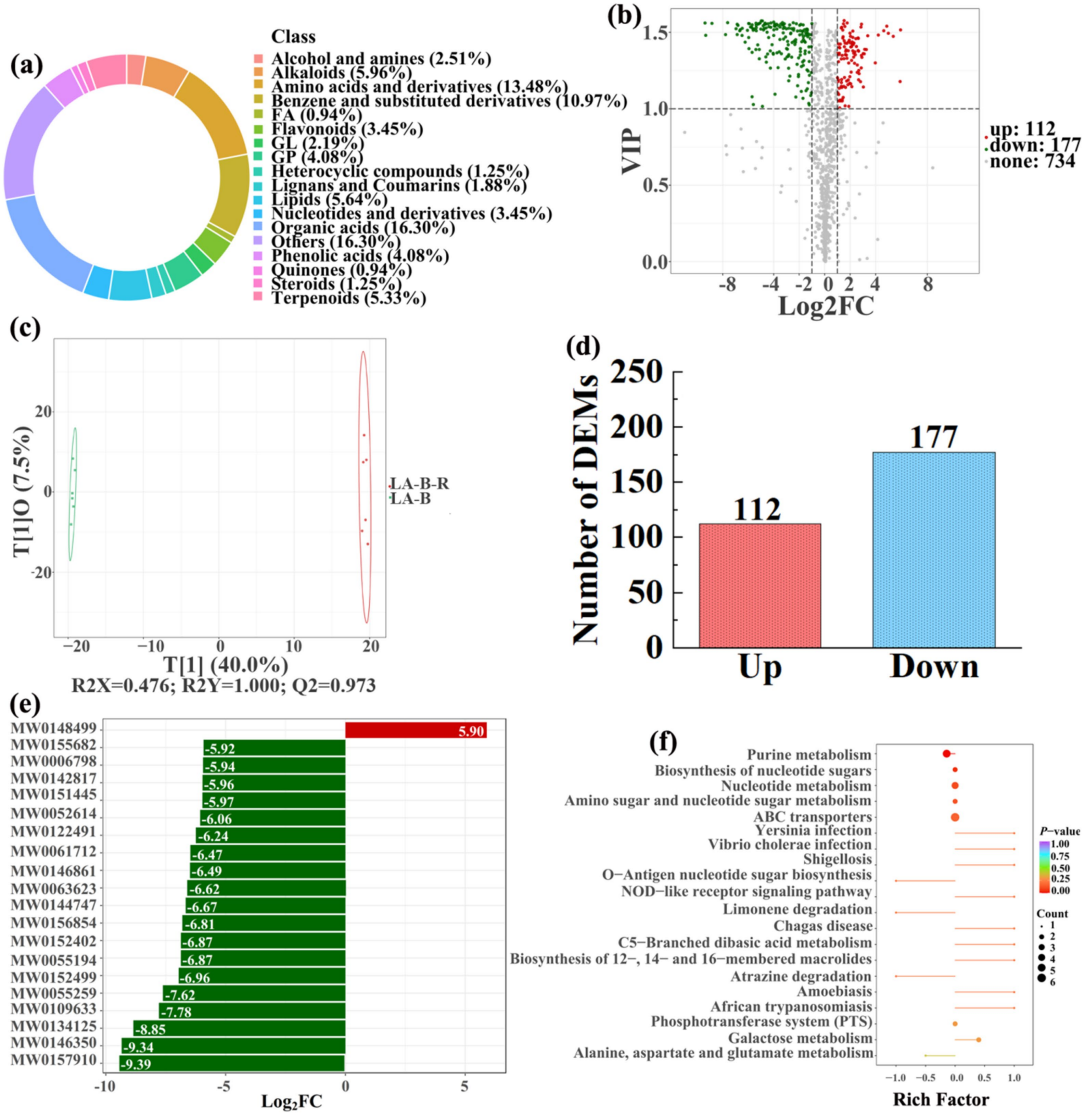
Donut plots showing metabolite classification and proportion **(a)**, volcano plot **(b)**, orthogonal projection to latent structures-discriminant analysis (OPLS-DA) score map **(c)**, the number of DEMs **(d)**, top 20 DEMs with largest VIP **(e)**, KEGG enrichment analysis of differential soil metabolites in conversion of bamboo garden to paddy field (LA-B-R) *vs* bamboo garden (LA-B) **(f)**.

**Figure 13:**
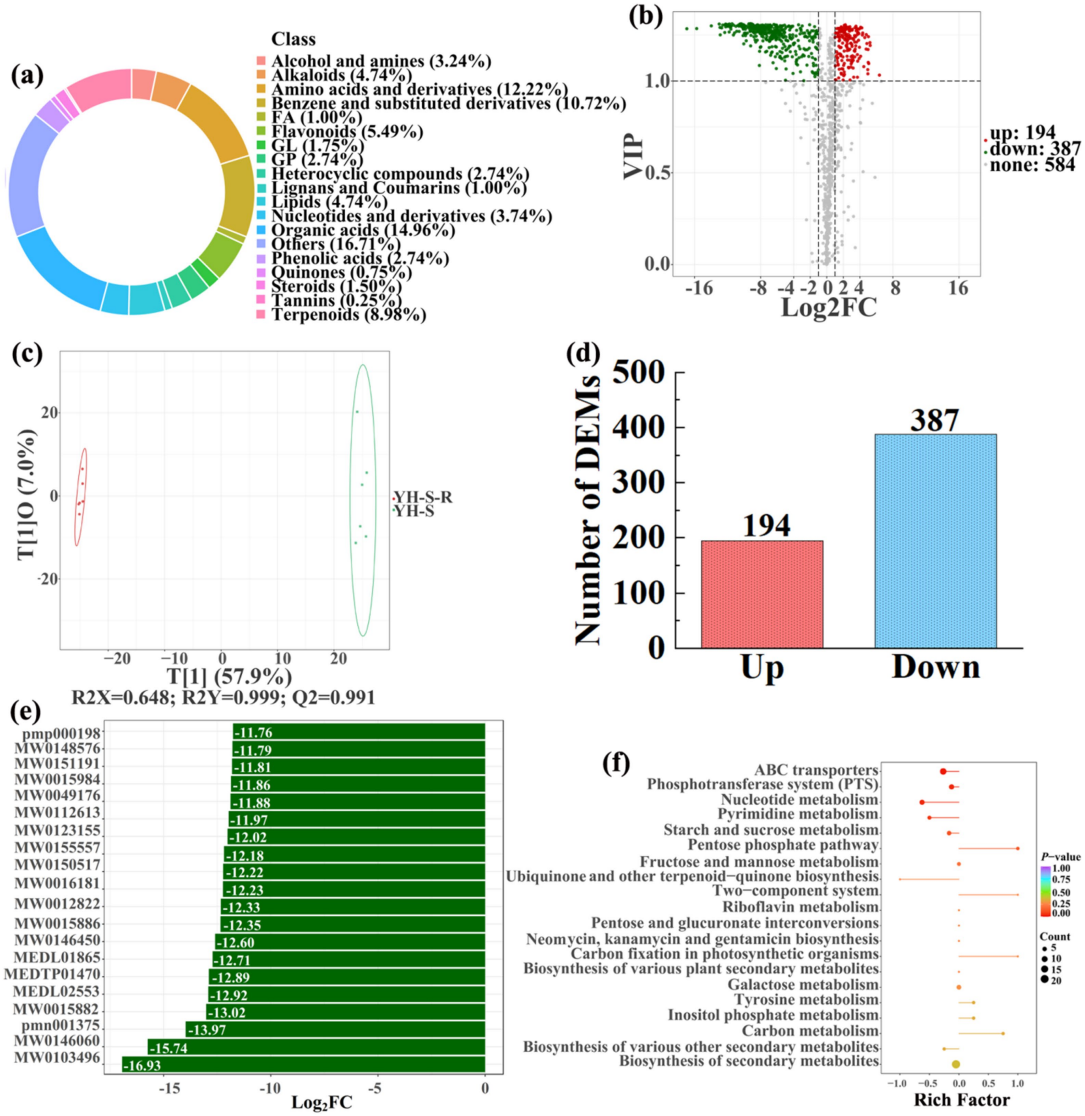
Donut plots showing metabolite classification and proportion **(a)**, volcano plot **(b)**, orthogonal projection to latent structures-discriminant analysis (OPLS-DA) score map **(c)**, the number of DEMs **(d)**, top 20 DEMs with largest VIP **(e)**, KEGG enrichment analysis of differential soil metabolites in conversion of nursery stock base to paddy field (YH-S-R) *vs* nursery stock base (YH-S) **(f)**.

To analyze and compare the changes in the soil metabolites in the paddy fields converted from non-grain cultivated lands, LC–MS analysis was performed, while OPLS-DA was used to construct a score map of metabolites to identify variables differed between different treatments. Results revealed that the distribution of soil metabolites could be effectively distinguished between the non-grain cultivated lands and the converted paddy fields, as reflected by the sample distributions of each group in the positive and negative directions of t[1], with corresponding model values of *R^2^X* (cum) = 0.446–0.648, *R^2^Y* (cum) = 0.999–1.000, and *Q^2^* (cum) = 0.951–0.991. Furthermore, this inference could also be confirmed by volcano plot (based on VIP > 1 and *p* < 0.05) (Figures 8–13). A total of 5,827 metabolites were obtained from all groups, which mainly referred to amino acids and derivatives (9.76–13.78%), benzene and substituted derivatives (9.27–14.14%), flavonoids (3.45–8.54%), lipids (3.02–9.22%), organic acids (12.12–16.30%), terpenoids (4.27–9.58%), and so on. Furthermore, enrichment analysis of the KEGG pathway indicated that these DEMs might be associated with ABC transporters, biosynthesis of cofactors, biosynthesis of secondary metabolites, fructose and mannose metabolism, metabolic pathways, microbial metabolism in diverse environments, nucleotide metabolism, phosphotransferase system, purine metabolism, starch and sucrose metabolism, consequently playing a significant role during conversation of non-grain cultivated land to paddy field.

In detail, 304 out of 852 identified metabolites were differentially expressed by conversion of loquat garden to paddy field, while the top 20 DEMs (5 upregulation and 15 downregulation) with largest VIP were visualized by bar chart (Figure 8), 305 out of 1,021 identified metabolites were differentially expressed by conversion of mulberry field to paddy field, while the top 20 DEMs with largest VIP were all downregulated (Figure 9), 288 out of 874 identified metabolites were differentially expressed by conversion of blueberry garden to paddy field, while 5 upregulation and 15 downregulation were found in the top 20 DEMs with largest VIP (Figure 10), 437 out of identified 892 metabolites were differentially expressed by conversion of vineyard to paddy field, while 9 upregulation and 11 downregulation were found in the top 20 DEMs with largest VIP (Figure 11), 289 out of identified 1,023 metabolites were differentially expressed by conversion of bamboo garden to paddy field, while 1 upregulation and 19 downregulation were found in the top 20 DEMs with largest VIP (Figure 12), 581 out of 1,165 identified metabolites were differentially expressed by conversion of nursery stock base to paddy field, while top 20 DEMs with largest VIP were all downregulated (Figure 13). Across all six different land-use conversions, amino acids and derivatives, benzene and substituted derivatives, heterocyclic compounds, organic acids, and terpenoids were the most consistently differentially expressed metabolite classes (Table 4). The functions of these DEMs were determined by the KEGG pathway analysis (Figures 8–13f). In contrast with non-grain cultivated land, the distinct metabolites mostly pertain to “microbial metabolism in diverse environments” and “ABC transporters” in paddy field converted from loquat garden; “biosynthesis of secondary metabolites” and “microbial metabolism in diverse environments” in paddy field converted from mulberry field; “microbial metabolism in diverse environments” and “biosynthesis of cofactors” in paddy field converted from blueberry garden; “metabolic pathways” and “microbial metabolism in diverse environments” in paddy field converted from vineyard; “ABC transporters,” “purine metabolism,” and “nucleotide metabolism” in paddy field converted from bamboo garden; “biosynthesis of secondary metabolites” and “ABC transporters” in paddy field converted from nursery stock base. An overlapping enrichment of “microbial metabolism in diverse environments” and “ABC transporters” in most paddy field converted from non-grain cultivated land suggests that the two specific metabolic pathways are vital for their survival and ecological niche establishment.

**Table 4:** Top 20 DEMs with largest VIP under different groups.

Groups	Index	Compounds	Class	Regulated	Most consistent DEMs
JD-L *vs* JD-L-R	MW0153324	(1S,2R)-5,7,8-trimethoxy-2,3-dimethyl-1-(2,4,5-trimethoxyphenyl)-1,2-dihydronaphthalene	Lignans and Coumarins	Up	Amino acids and derivatives, Organic acids, Terpenoids
	MW0000104	7-Ethyl-10-(4-N-aminopentanoic acid)-1-piperidino)carbonyloxycamptothecin	Alkaloids	Up	
	MW0156550	Saupirin	Terpenoids	Up	
	MW0062077	Pisumionoside	Terpenoids	Up	
	MW0109227	Phenylalanylserine	Amino acids and derivatives	Up	
	MW0016281	N-octanoylsphingosine 1-phosphate	Organic acids	Down	
	MW0127150	{[5-(2-Furyl)-1,3,4-oxadiazol-2-yl]thio}acetic acid	Organic acids	Down	
	MW0145374	Arg-His-His	Amino acids and derivatives	Down	
	MW0011981	10,12-Tricosadiynoic acid	Lipids	Down	
	MEDN1026	Biosone	Terpenoids	Down	
	MW0145690	Asn-Asn-Leu-Asn-Val	Amino acids and derivatives	Down	
	MW0112903	5-O-caffeoyl-4-O-sinapoylquinic acid	Organic acids	Down	
	MW0166457	Guaiacol beta-primeveroside	Alcohol and amines	Down	
	MW0016181	Betulonic acid	Terpenoids	Down	
	MW0113885	Aloesin	Others	Down	
	MW0151584	Ile-Ser-Thr-Glu	Amino acids and derivatives	Down	
	MEDL02042	Cianidanol	Flavonoids	Down	
	MW0008260	N-Caffeoylputrescine	Alkaloids	Down	
	MW0154943	Pavoninin-1	Steroids	Down	
	MEDL02600	Tomentosolic acid	Terpenoids	Down	
CA-M *vs* CA-M-R	MW0139564	trans-Resveratrol 4’-O-glucuronide	Others	Down	
	MW0144049	8-Epideoxyloganin	Others	Down	Alkaloids, Amino acids and derivatives, Benzene and substituted derivatives, Organic acids
	MW0160123	Benzyl beta-primeveroside	Phenolic acids	Down	
	MW0130200	5,7-Dihydroxy-3,6,8,3′,4′-pentamethoxyflavone	Flavonoids	Down	
	MW0151006	His-Lys-Asn	Amino acids and derivatives	Down	
	MW0148857	Echimidine	Alkaloids	Down	
	MW0124227	Garcinone E	Flavonoids	Down	
	MW0015241	7alpha-Thiomethylspironolactone	Others	Down	
	MW0137975	Dihydromunduletone	Others	Down	
	MW0131715	3,4,5-Trihydroxy-6-[[3,4,5-trihydroxy-6-[[2-(2-hydroxypropan-2-yl)-7-oxo-2,3-dihydrouro[3,2-g]chromen-9-yl]oxy]oxan-2-yl]methoxy]oxane-2-carboxylic acid	Organic acids	Down	
	MW0016972	Cellobiotol	Steroids	Down	
	MW0164056	(1S,2R,3S,4S,5R,6S)-4-azaniumyl-2-{[azaniumyl(imino)methyl]amino}-3,5,6-trihydroxycyclohexyl phosphate	Others	Down	
	MW0146643	4-deoxy-alpha-L-threo-hex-4-enopyranuronosyl-(1- > 4)-beta-D-glucopyranosyl-(1- > 4)-alpha-L-rhamnopyranosyl-(1- > 3)-D-glucopyranose	Others	Down	
	MW0131629	3,4,5-trihydroxy-6-(2-hydroxy-5-{3,5,6,7-tetrahydroxy-8-[3,4,5-trihydroxy-6-(hydroxymethyl)oxan-2-yl]-3,4-dihydro-2H-1-benzopyran-2-yl}phenoxy)oxane-2-carboxylic acid	Organic acids	Down	
	MW0013427	butenyl]-2-cyclohexene-1-one	Others	Down	
	ZINC85921668	Thelephantin L	Benzene and substituted derivatives	Down	
	MW0137269	Apiosylskimmin	Lignans and Coumarins	Down	
	MW0139314	Petunidin 3-galactoside	Flavonoids	Down	
	MW0006499	Carboxytolbutamide	Benzene and substituted derivatives	Down	
	MW0132475	3,4,5-trihydroxy-6-{4-[(E)-2-(3,4,5-trihydroxyphenyl)ethenyl]phenoxy}oxane-2-carboxylic acid	Organic acids	Down	
TL-B *vs* TL-B-R	MW0153058	Lys-Lys-Gly-Ala-Glu	Amino acids and derivatives	Up	Amino acids and derivatives, Benzene and substituted derivatives, Heterocyclic compounds, Organic acids, Terpenoids
	MW0138535	Isorhapontin	Heterocyclic compounds	Up	
	MW0158567	Tyr-Phe-Glu-Lys	Amino acids and derivatives	Up	
	MEDL02225	DL-Arabinose	Others	Up	
	MW0110027	2-(2-(2-Methoxyethoxy)ethoxy)ethyl methacrylate	Others	Up	
	MW0113064	NCGC00380744-01_C22H32O11_beta-D-Glucopyranoside, 4-hydroxy-3-(3-methyl-2-buten-1-yl)phenyl 6-O-[(2R,3R,4R)-tetrahydro-3,4-dihydroxy-4-(hydroxymethyl)-2-furanyl]-	Benzene and substituted derivatives	Down	
	MW0131213	3-(3,7-dimethylocta-2,6-dien-1-yl)-5,7-dihydroxy-6-(3-methylbut-2-en-1-yl)-2-(2,4,5-trihydroxyphenyl)-3,4-dihydro-2H-1-benzopyran-4-one	Others	Down	
	MW0113760	NCGC00380161-01_C20H28O12_6-O-(Phenylacetyl)-alpha-D-glucopyranosyl alpha-D-glucopyranoside	Benzene and substituted derivatives	Down	
	MW0161370	Zeaxanthin glucoside	Alcohol and amines	Down	
	MW0165028	Precorrin-7(6-)	Others	Down	
	MW0145407	Arg-Leu-Arg-Glu-Lys	Amino acids and derivatives	Down	
	MW0112615	3,4,5-trihydroxy-6-{[3-hydroxy-2-(hydroxymethyl)-2-methylpropanoyl]oxy}oxane-2-carboxylic acid	Organic acids	Down	
	MW0009218	Benzenesulfonic acid, undecyl-	Benzene and substituted derivatives	Down	
	MW0155768	Pro-Ala-Leu-Phe-Leu	Amino acids and derivatives	Down	
	MW0158716	TyrMe-TyrMe-OH	Amino acids and derivatives	Down	
	MW0143349	1,3,4,10,11,12-Hexahydroxy-6-methyltetracene-2-carboxamide	Alcohol and amines	Down	
	MEDP1337	LysoPC 18:0	Lipids	Down	
	MEDL02585	Veranisatin B	Terpenoids	Down	
	ZINC95910020	Carvacrol 2-O-beta-glucopyranosyl(1–2)-beta-glucopyranoside	Alcohol and amines	Down	
	MW0005107	4-hydroxylamino-2,6-dinitrotoluene	Benzene and substituted derivatives	Down	
FY-G *vs* FY-G-R	MW0128017	Catechin tetramethylether	Benzene and substituted derivatives	Up	Amino acids and derivatives, Benzene and substituted derivatives, Heterocyclic compounds, Organic acids, Terpenoids
	MW0148974	Eremantholide A	Heterocyclic compounds	Up	
	MW0105039	3-Hydroxydodecanedioic acid	Heterocyclic compounds	Up	
	MW0111410	(2R)-2-Hydroxy-2-(4-hydroxyphenyl)ethyl glucosinolate	Others	Up	
	MW0015367	Tetranor 12-HETE	Others	Up	
	MW0159026	Val-His-Leu-Asp	Amino acids and derivatives	Up	
	MW0144594	Ala-His-Leu-Asp	Amino acids and derivatives	Up	
	MW0104771	2-Hydroxydecanedioic acid	Organic acids	Up	
	MW0107641	L-Acetopine	Amino acids and derivatives	Up	
	MW0103537	2′,3’-Dideoxycytidine 5′-triphosphate	Nucleotides and derivatives	Down	
	MW0144629	Ala-Leu-Ala-Pro-Lys	Amino acids and derivatives	Down	
	MW0124558	JWH 018 N-pentanoic acid metabolite-d4	Organic acids	Down	
	MW0005550	5-Amino-2-(p-toluidino)benzenesulphonic acid	Benzene and substituted derivatives	Down	
	MW0002646	2-amino-4-(4-nitrophenyl)-5-oxo-7-phenyl-5,6,7,8-tetrahydro-4H-chromen-3-yl cyanide	Benzene and substituted derivatives	Down	
	MW0127287	3-(Ethylthio)-1,2,4-thiadiazol-5-amine	Heterocyclic compounds	Down	
	MW0114361	D-Ribulose 1,5-bisphosphate	Others	Down	
	MW0061034	(4Ar,6aS,9aR)-1,8,8-trimethyl-2-oxo-1,4,4a,6a,7,9-hexahydropentaleno[1,6a-c]pyran-5-carboxylic acid	Terpenoids	Down	
	MW0160932	threo-3-methyl-L-aspartate(2-)	Organic acids	Down	
	MW0148006	Cys-Glu-His	Amino acids and derivatives	Down	
	MW0128216	(E)-1,7-bis(4-hydroxyphenyl)hept-4-en-3-one	Others	Down	
LA-B *vs* LA-B-R	MW0148499	Dihydrocorynantheine	Alkaloids	Up	Amino acids and derivatives, Benzene and substituted derivatives, Heterocyclic compounds, Terpenoids
	MW0155682	Prechromomycin B	Benzene and substituted derivatives	Down	
	MW0006798	Dicyclohexyl phthalate	Benzene and substituted derivatives	Down	
	MW0142817	3beta-(1-Pyrrolidinyl)-5alpha-pregnane-11,20-dione	Others	Down	
	MW0151445	Ile-His-Arg-Arg	Amino acids and derivatives	Down	
	MW0052614	Erinacine P	Heterocyclic compounds	Down	
	MW0122491	7-Hydroxymethotrexate	Heterocyclic compounds	Down	
	MW0061712	PI(18:0/20:4(5Z,8Z,11Z,14Z))	GP	Down	
	MW0146861	Brevetoxin A	Heterocyclic compounds	Down	
	MW0063623	Sphingosine 1-phosphate(d19:1-P)	SL	Down	
	MW0144747	Ala-Thr-Ile-Lys	Amino acids and derivatives	Down	
	MW0156854	Ser-Lys-Val-Glu	Amino acids and derivatives	Down	
	MW0152402	Leu-Leu-Lys-Gln-Gly	Amino acids and derivatives	Down	
	MW0055194	N-(1,3-Dihydroxyoctadecan-2-YL)-6-[(7-nitro-2,1,3-benzoxadiazol-4-YL)amino]hexanamide	SL	Down	
	MW0152499	Leu-Ser-Pro-Lys-Lys	Amino acids and derivatives	Down	
	MW0055259	3-[6-[(E)-4,6-dimethyloct-2-en-2-yl]-5-methyloxan-2-yl]-4-hydroxy-5-(4-hydroxyphenyl)-1H-pyridin-2-one	Terpenoids	Down	
	MW0109633	Ser-Pro-Lys	Amino acids and derivatives	Down	
	MW0134125	5,7-Dihydroxy-2-phenyl-8-[3,4,5-trihydroxy-6-(hydroxymethyl)oxan-2-yl]-6-(3,4,5-trihydroxyoxan-2-yl)chromen-4-one	Others	Down	
	MW0146350	Asp-HoPhe-OH	Amino acids and derivatives	Down	
	MW0157910	Thr-Ser-Lys	Amino acids and derivatives	Down	
YH-S *vs* YH-S-R	pmp000198	Soyasaponin betac	Terpenoids	Down	Amino acids and derivatives, Heterocyclic compounds, Organic acids, Terpenoids
	MW0148576	Dioncophylline C	Heterocyclic compounds	Down	
	MW0151191	Hoiamide A	Alcohol and amines	Down	
	MW0015984	Astragaloside IV	Terpenoids	Down	
	MW0049176	DEHYDRO(11,12)URSOLIC ACID LACTONE	Others	Down	
	MW0112613	3,4,5-trihydroxy-6-{[3-hydroxy-10-(3-hydroxybutanoyl)-2,2-dimethyl-8-oxo-6-propyl-2H,3H,4H,8H-pyrano[3,2-g]chromen-5-yl]oxy}oxane-2-carboxylic acid	Organic acids	Down	
	MW0123155	Brazilin	Flavonoids	Down	
	MW0155557	PI 18:2	GP	Down	
	MW0150517	Gly-Tyr-Ile-Ser-Ala	Amino acids and derivatives	Down	
	MW0016181	Betulonic acid	Terpenoids	Down	
	MW0012822	Azelaoyl PAF	GP	Down	
	MW0015886	Araloside A	Terpenoids	Down	
	MW0146450	Avenacin A-1	Alkaloids	Down	
	MEDL01865	Phytolaccoside D	Terpenoids	Down	
	MEDTP01470	Octyl-Beta-D-Glucopyranoside	Others	Down	
	MEDL02553	Piperine	Alkaloids	Down	
	MW0015882	Araliasaponin IV	Terpenoids	Down	
	pmn001375	1-Hydroxypinoresinol-1-O-Glucoside	Lignans and Coumarins	Down	
	MW0146060	Asp-Asp-Ser	Amino acids and derivatives	Down	
	MW0103496	cis-Zeatin riboside	Nucleotides and derivatives	Down	

### 3.4 Correlations among soil properties, bacteria, and metabolites

**Figure 14:**
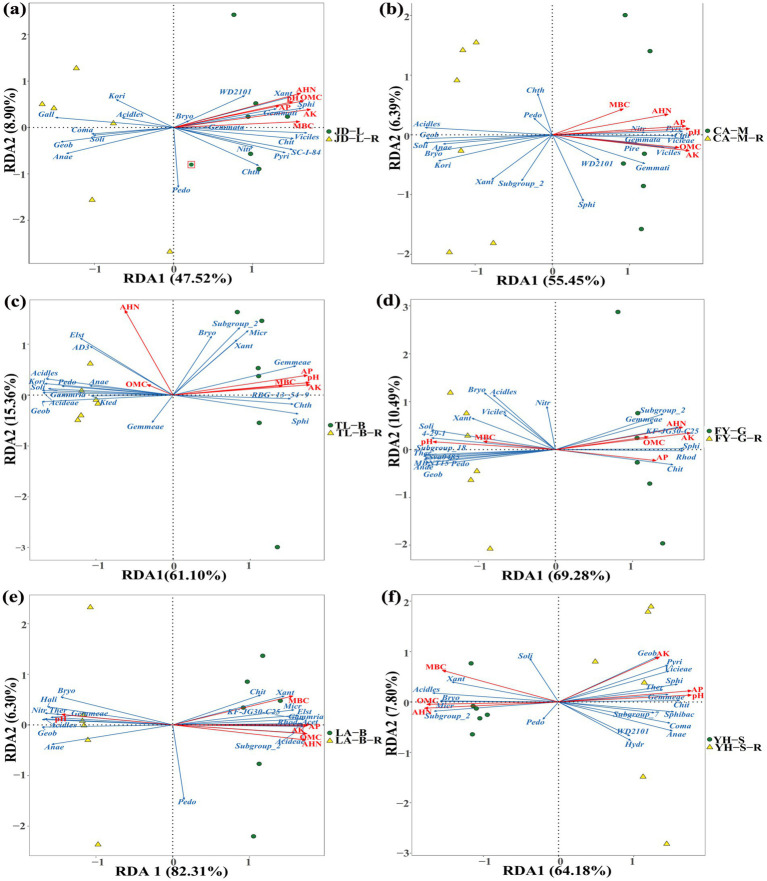
Redundancy discriminant analysis (RDA) of the root-zone soil bacterial community compositions at the family level with soil properties. The conversion of loquat garden to paddy field **(a)**, conversion of mulberry field to paddy field **(b)**, conversion of blueberry garden to paddy field **(c)**, conversion of vineyard to paddy field **(d)**, conversion of bamboo garden to paddy field **(e)**, and conversion of nursery stock base to paddy field **(f)**. *Acet*, *Acetobacteraceae*; *Acideae*, *Acidobacteriaceae*; *Acidles*, *Acidobacteriales*; *Anae*, *Anaerolineaceae*; *Bryo*, *Bryobacteraceae*; *Chit*, *Chitinophagaceae*; *Chth*, *Chthoniobacteraceae*; *Coma*, *Comamonadaceae*; *Elst*, *Elsterales*; *Gall*, *Gallionellaceae*; *Geob*, *Geobacteraceae*; *Gemmata*, *Gemmataceae*; *Gemmati*, *Gemmatimonadaceae*; *Gammria*, *Gammaproteobacteria*; *Hali*, *Haliangiaceae*; *Hydr*, *Hydrogenophilaceae*; *Kori*, *Koribacteraceae*; *Kted*, *Ktedonobacteraceae*; *Micr*, *Micropepsaceae*; *Nitr*, *Nitrosomonadaceae*; *Pedo*, *Pedosphaeraceae*; *Pire*, *Pirellulaceae*; *Pyri*, *Pyrinomonadaceae*; *Rhod*, *Rhodanobacteraceae*; *Soli*, *Solibacteraceae*; *Sphi*, *Sphingomonadaceae*; *Sphibac*, *Sphingobacteriaceae*; *Ther*, *Thermodesulfovibrionia*; *Vicieae*, *Vicinamibacteraceae*; *Viciles*, *Vicinamibacterales*; *Xant*, *Xanthobacteraceae*. SOM, organic matter contains; AHN, alkaline hydrolysis N; AP, available P; AK, available K; MBC, microbial biomass carbon. Arrows indicate the direction and magnitude of soil properties (pH, SOM, AHN, AP, AK, and MBC) associated with the different bacteria.

RDA based on family-level abundances was carried out to examine the correlation between environmental factors and soil bacterial communities, and the results showed that a total of 56.42–88.61% of the cumulative variance of bacterial community-factor correction occurred at the family level (Figure 14; Table 5). In detail, the contributions of the 6 variables were AK (*F* = 73.54–97.78%, *p* = 0.001–0.04), AHN (*F* = 70.70–96.49%, *p* = 0.001–0.008), pH (*F* = 65.39–97.31%, *p* = 0.001–0.017), AP (*F* = 53.27–99.02%, *p* = 0.001–0.037), MBC (*F* = 27.12–82.86%, *p* = 0.002–0.229), and SOM (*F* = 4.75–94.83%, *p* = 0.001–0.818) during conversion of non-grain cultivated land to paddy field. Thus, it can be inferred that AK, AHN, pH, and AP were the primary factors associated with bacterial community variation, suggesting that soil nutrient elements clearly affected bacterial family-level distributions.

**Table 5:** Contribution of the soil environment to bacterial taxa at the family level under different groups.

Soil environment	Contribution at the bacterial family level (%)					
	JD	CA	TL	FY	LA	YH
pH	70.09	96.41	97.31	77.70	65.39	94.76
SOM	90.87	82.64	4.75	46.45	94.83	92.91
AHN	91.92	70.70	95.01	89.55	94.68	96.49
AP	60.03	91.11	97.18	53.27	99.02	95.46
AK	95.42	97.21	97.47	97.78	94.10	73.45
MBC	78.29	29.64	62.38	27.12	82.86	82.12

SOM, soil organic matter; AHN, alkaline hydrolysis nitrogen; AP, available phosphorus; AK, available potassium; MBC, microbial biomass carbon.

**Figure 15:**
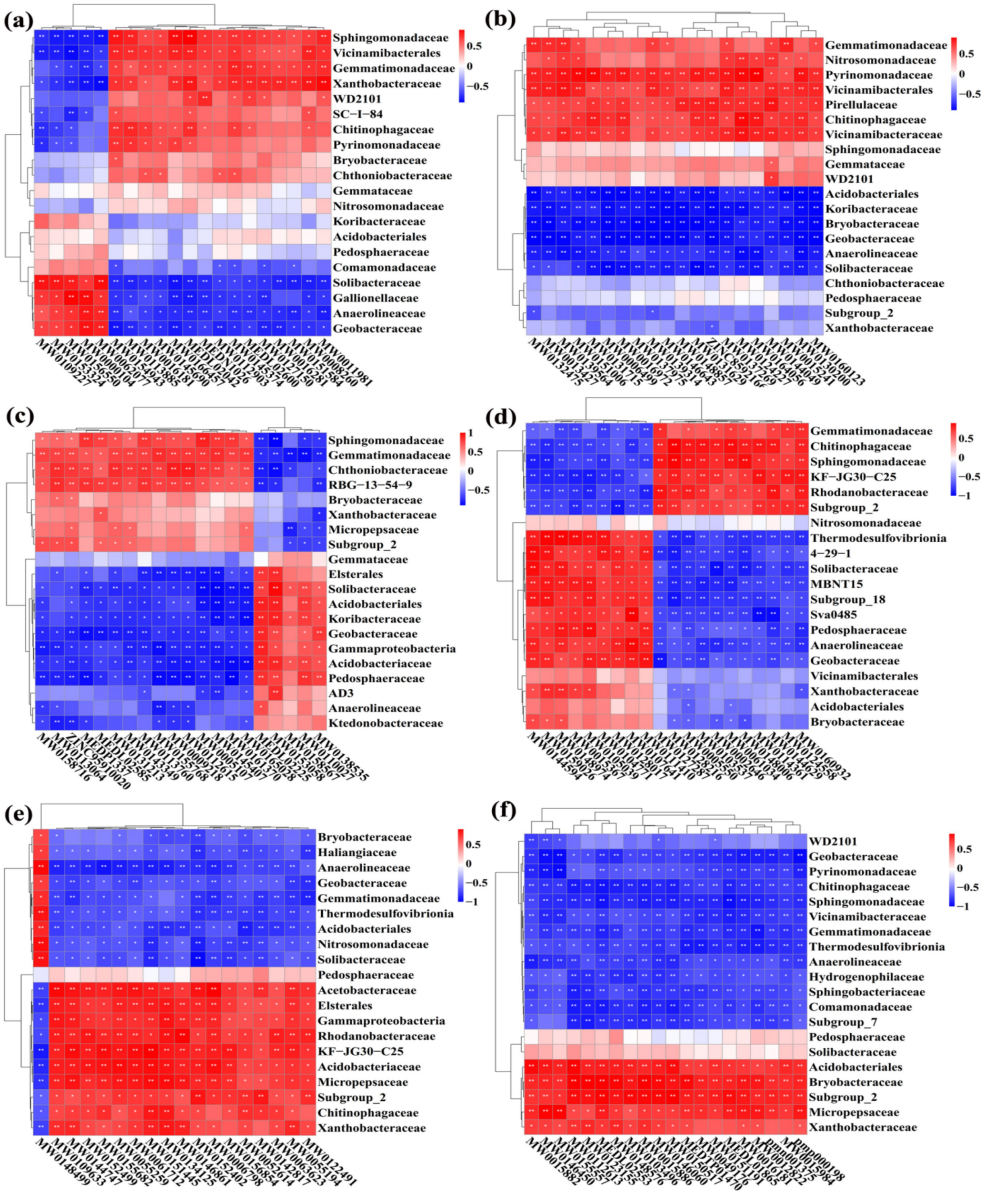
Correlation heat map between the top 20 family of bacteria and significant DEMs under different groups. The conversion of loquat garden to paddy field **(a)**, conversion of mulberry field to paddy field **(b)**, conversion of blueberry garden to paddy field **(c)**, conversion of vineyard to paddy field **(d)**, conversion of bamboo garden to paddy field **(e)**, and conversion of nursery stock base to paddy field **(f)**. *Indicated a significant correlation at *p* < 0.05, **indicated a significant correlation at *p* < 0.01.

To further analyze the correlation relationship between bacteria and metabolites, the clustering heat map was drawn based on bacterial top 20 family and 20 DEMs with largest VIP (Figure 15). Indeed, the top 20 upregulated DEMs were significantly correlated with 15 bacteria (except Acidobacteriales, Gemmataceae, Koribacteraceae, Nitrosomonadaceae, and Pedosphaeraceae) in the paddy fields converted from loquat garden, 17 bacteria (except Chthoniobacteraceae, Pedosphaeraceae and Sphingomonadaceae) in the paddy fields converted from mulberry field, 19 bacteria (except Gemmataceae) in the paddy fields converted from blueberry garden, 18 bacteria (except Nitrosomonadaceae and Vicinamibacterales) in the paddy fields converted from vineyard, 19 bacteria (except Pedosphaeraceae) in the paddy fields converted from bamboo garden, 18 bacteria (except Pedosphaeraceae and Solibacteraceae) in the paddy fields converted from nursery stock base, respectively (Table 4).

Taken together, the DEMs (amino acids and derivatives, organic acids, benzene and substituted derivatives, heterocyclic compounds, and terpenoids) of root-zone soils could significantly alter the soil bacteria. Especially, they were significantly negative correlated with the two key families of Anaerolineaceae and Geobacteraceae, while the two families were also negative correlated with AP and AK. In other word, the lower AP, AK, and DEMs thereby could help in the coordination of the root-zone bacteria during conversion of non-grain cultivated land to paddy field. Previous research reported that soil moisture regimes influenced bacterial community structure both directly and indirectly by changing nutrient availability and oxygen concentrations, and flooding with less oxygen availability promoted the growth of facultative anaerobic bacteria, such as Anaerolineaceae (with the ability to providing organic acid such as acetate to other microbes) and Geobacteraceae (the main ferric reducers in anaerobic environments) (Liang et al., 2015; Wang et al., 2020).

## 4 Discussion

Cultivated land is paramount for grain production, serving as a vital strategic resource worldwide. However, due to the pursuit for economic benefits, numerous cultivated lands have been used for non-grain production, thus leading to significant impacts on the arable land-use structure and ecosystem function (Su et al., 2020). Soil microbes mainly including bacteria, fungi, archaea play an essential role in soil ecosystem by driving soil functional process including nutrient cycling, organic matter transformation, plant disease control, and plant productivity promotion (Philippot et al., 2013; Ling et al., 2014; Finzi et al., 2015; Pieterse et al., 2016). Among them, soil bacteria are essential members of soil microbial community (He et al., 2023), and presented larger environmental niche breadths than fungi and archaea (Malard et al., 2022). However, soil interference (such as changes in land use, vegetation, soil fertility, pH) can significantly affect soil bacteria in their taxonomy and functionality (Liang and Geng, 2023; Cordovez et al., 2019; Samaddar et al., 2019). In addition, soil bacteria are related to soil metabolites (Song et al., 2020). Here, the combined analyses of the soil physicochemical properties, bacterial community structure, and metabolite were able to explore the influence mechanism of non-grain production on cultivated land, which will in guiding the conversion of non-grain cultivated land to paddy field.

The soil properties in the non-grain cultivated land were significantly affected by the conversion to paddy field, and the impact depended on the soil parameters and the type of non-grain crops. In general, conversion of non-grain cultivated land to paddy field caused a remarkable decrease in the concentration of soil nutrients, while there were some recovery trajectories of soil nutrients in the vineyard or nursery stock conversions. Indeed, results revealed that the soil pH can be significantly reduced in the paddy fields converted from loquat garden, mulberry field, and blueberry garden, but improved from vineyard, bamboo garden, and nursery stock base. The SOM, AHN, AP (except nursery stock base), AK (except nursery stock base), and MBC (except vineyard) can be significantly reduced by the conversion of non-grain cultivated land to paddy fields, which may be due to the excessive use of fertilizer in non-grain cultivated lands. For example, the quantity of fertilizer usage on flowers and fruit trees was 3.85 fold and 2.45 fold of grain crops, respectively (Wang et al., 2018). Vegetable production systems had higher N (264.3 kg/ha) and P (101.0 kg/ha) fertilizer input than rice cultivation (Wang et al., 2019). Broadly, farmers were inclined to apply more fertilizer on dry land than in paddy fields (Jiao et al., 2017), while fertilization with N and P could increase soil organic carbon, N, and P concentrations (Wang N. et al., 2024). However, long-term overuse of chemical fertilizers could alter the soil pH, increase acidification, decrease soil quality (Pahalvi et al., 2021), while conversion of upland crop cultivation to paddy rice is an essential methodology to improving ecology (Lü et al., 2017). Therefore, it can be inferred that it is beneficial for soil sustainable ecological function restoration by conversion of non-grain cultivated land to paddy field.

Following the measurement of the bacterial community diversity in all soil samples using 16S rRNA gene high-throughput sequencing, we found that the bacterial OTUs number and Chao1 index was increased by conversion of non-grain cultivated land to paddy field (except blueberry garden). These results suggest that conversion of non-grain cultivated land to paddy field had the distinct impact in increasing the robustness of bacterial communities. Further PCA assay also revealed that there were significant differences in the root-zone soil bacterial communities between non-grain cultivated lands and the paddy fields converted from the corresponding non-grain cultivated lands. Liang and Geng (2023) showed that non-grainization consolidation by conversion of dryland to paddy field enhanced the α-diversity content (including Ace, Chao1, Coverage, and Shannon indices) in terms of soil bacterial community diversity. Indeed, agricultural land consolidation that widely applied in farmland improvement has been found to be able to significantly impact soil microbial community diversity and composition (Lin et al., 2020). In agreement with previous reports, conversion of non-grain cultivated land to paddy field also caused the alteration of some certain microbes, thus reshaping the root-zone soil bacterial community of rice field converted from non-grain cultivated land.

At the phylum level, Chloroflexi, Desulfobacterota, and Nitrospirota were significantly increased in most of the paddy fields converted from non-grain cultivated lands. At the family level, conversion of non-grain cultivated land to paddy field could significantly enrich Anaerolineaceae, Bryobacteraceae, Comamonadaceae, Gallionellaceae, Geobacteraceae, Haliangiaceae, Hydrogenophilaceae, Koribacteraceae, Ktedonobacteraceae, MBNT15, Nitrosomonadaceae, Pedosphaeraceae, Solibacteraceae, Subgroup_7, Subgroup_18, Sva0485, 4-29-1, Thermodesulfovibrionia, and WD2101. Furthermore, LEfSe also obtained 48 bacterial biomarkers in all treatments between different groups. In particular, conversion of six non-grain cultivated lands to paddy field in this study resulted in enrichment of some bacteria including Desulfobacterota (1.26–21.50 fold), Nitrospirota (4.29–14.54 fold), and Chloroflexi (0.81–3.08 fold), which might have important functional implications on rice growth, and could be used as potential biomarkers for successful land restoration.

In agreement with the result of this study, Chloroflexi and Nitrospirota play important roles in the nitrification process in the soil nitrogen cycle by oxidizing nitrite to nitrate (Prabhu et al., 2022). Desulfobacterales plays an important role in nitrogen cycling and contributes 12% of the genes of nitrogen pathways on average (Nie et al., 2021). Anaerolineaceae can be used as abundant primary fermenters in anaerobic digesters, and has the ability of providing organic acid such as acetate to other microbes (Liang et al., 2015; Mcllroy et al., 2017). Geobacteraceae is an important dissimilatory Fe(III) reducer that affects the cycles of multiple elements, while dissimilatory iron reduction mediated by the Geobacteraceae may influence the rice yields by affecting the biogeochemical cycles of nutrient elements (Li et al., 2020). Nitrosomonadaceae plays a key role in the nitrogen cycle (Prosser et al., 2014). Pedosphaeraceae can detoxify arsenic and antimony (Huang et al., 2014). Obviously, these bacteria may have significant potential in colonizing and altering soil fertility in paddy fields converted from non-grain cultivated land by enhancing nutrient uptake, improving soil conditions and increasing bioavailability. On the other hand, this study focuses on bacteria due to it is the most major kingdom in soil, however, it will capture a more holistic soil microbiome profile by including fungal or archaeal community analyses.

Moreover, network assay can reveal the co-occurrence patterns between soil microbial members and complex associations within soil microbial communities (Wang J. et al., 2022). The co-occurrence networks constructed in this study indicated that the number of network nodes and edges were higher in non-grain cultivated lands (except loquat garden and nursery stock base) compared to the paddy fields converted from the corresponding non-grain cultivated land. Conversely, the modularity of networks was higher in the converted paddy fields than that of from the corresponding non-grain cultivated land. Due to more nodes and edges indicating a more complex network structure, while high modularity representing high structural stability of network (Freundt, 2021; Ma et al., 2021), thus, it can be inferred that conversion of non-grain cultivated land to paddy fields is good for stability of soil bacterial community. Align with this study, Cornell et al. (2023) also showed that land use conversion in a temperate grassland increased network complexity and stability of soil microbial communities, which have been reported to be highly associated with soil ecosystem function.

The variability within each treatment was the main challenges in comparing metabolomic profiles across such ecologically distinct conversion types. In order to address the issue, soil sample in this study was collected by mixing a total of nine random soil cores, while the comparative analysis of metabolites was performed on each land type between the non-grain cultivated lands and the corresponding converted paddy fields. Generally, a sum of 5,827 metabolites were identified from all different groups, which were composed mainly of amino acids and derivatives, benzene and substituted derivatives, flavonoids, lipids, organic acids, terpenoids, with 794 upregulated and 1,410 downregulated metabolites. The OPLS-DA, volcano plot, and KEGG enrichment analysis showed that there was significant difference in the metabolite compositions between non-grain cultivated lands and the paddy fields converted from the corresponding non-grain cultivated land. Previous researches indicated that the differentially expressed metabolites were involved in different bio-activities and many bio-chemical activities in relation to rice growth and enhancement of soil fertility. For example, flavonoids play important roles in plant development and plant-environmental interactions (Dong and Lin, 2021), while lipids affect plant growth and development by involving cell membrane remodeling, anther fertility, seed formation, and response to adverse stresses (Wan et al., 2020).

Previous study also indicated that some secondary metabolism (such as flavonoids, terpenoids, strigolactones, and coumarins) could regulate the assembly of specific microbial taxa in the rhizosphere, while soil microbes can also enhance the promotion or inhibition of soil metabolites accumulation (Cheng et al., 2022; Wang L. et al., 2022). Finally, the regulation of key metabolites can increase crop yields in agroecosystems (Zhao et al., 2022). For example, flavoniods could regulate rhizosphere bacterial community structure (with a significant increase in the RA of Micrococcaceae and Nocardioidaceae) to enhance organic P mineralization, thereby facilitating P uptake and plant growth (Wang S. et al., 2024). In agreement with previous reports, the result of this study showed that amino acids and derivatives, organic acids, and terpenoids could recruit beneficial microbes, such as Anaerolineaceae and Geobacteraceae, to improve plant fitness and help plants cope with environmental changes during conversation non-grain cultivated land to paddy field. In other word, soil metabolites might play important roles in maintenance of soil ecosystem functions and crop yields during this conversation.

The correlation relationship between bacteria and DEMs in different group was determined by drawing the clustering he during conversation non-grain cultivated land to paddy field at map, which showed that the DEMs (amino acids and derivatives, organic acids, benzene and substituted derivatives, and other secondary metabolites) of root-zone soils were significantly positively or negatively correlated with the relative abundances of some bacteria, thereby helping in coordinating the root-zone bacteria during conversion of non-grain cultivated lands to paddy fields. Meanwhile, RDA in this study also indicated that soil pH, AK, AHN, and AP were the primary factors associated with bacterial community variation, showing the strongest predictive value for bacterial community shifts. For example, the two key families of Anaerolineaceae and Geobacteraceae in paddy field were negatively correlated with AP and AK. Therefore, these specific soil nutrients should be considered for land rehabilitation from non-grain land to paddy land. In agreement with the result of our study, Wan et al. (2023) and Li et al. (2024) also indicated that the growth of soil bacteria was often impacted by various environmental factors. Taken overall, this study revealed that good teamwork occurs among soil properties, bacteria, and metabolites during conversation of non-grain cultivated land to paddy field.

## 5 Conclusion

In conclusion, conversion of non-grain field to paddy field changed the soil properties, bacterial communities, and metabolites. Specifically, a higher OTUs number, a more diversity and stable bacterial community was obtained in paddy fields converted from non-grain fields than the corresponding non-grain fields. Furthermore, 48 bacterial biomarkers were identified across all different groups, with enriched abundances of Chloroflexi, Desulfobacterota, Nitrospirota, Anaerolineaceae, Geobacteraceae, Nitrosomonadaceae, and Pedosphaeraceae in converted paddy fields converted from non-grain cultivated lands, while 5,827 metabolites were identified in converted paddy fields and non-grain cultivated lands, with 794 of upregulation and 1,410 of downregulation. In addition, DEMs of root-zone soils were significantly correlated with bacteria, thereby helping in coordinating the root-zone bacteria during conversion of non-grain cultivated land to paddy field, while soil environmental properties (especially soil pH, AK, AHN, and AP) were related to variations in bacterial community composition. Overall, the result of this study indicated that the paddy fields converted from non-grain cultivated lands can be characterized by more richness, diversity and stable bacterial community structure, specific bacteria and metabolites, lower nutrition. Overall, this study provides a scientific basis and supporting evidence to explain the mechanism of conversion from non-grain cultivated lands to paddy fields, thus ensuring national food security.

## Data Availability

The datasets presented in this study can be found in online repositories. The names of the repository/repositories and accession number(s) can be found at: https://www.ncbi.nlm.nih.gov/, PRJNA1192420; https://db.cngb.org/, CNP0006336.
